# Multiple-Target Homotopic Quasi-Complete Path Planning Method for Mobile Robot Using a Piecewise Linear Approach

**DOI:** 10.3390/s20113265

**Published:** 2020-06-08

**Authors:** Gerardo Diaz-Arango, Hector Vazquez-Leal, Luis Hernandez-Martinez, Victor Manuel Jimenez-Fernandez, Aurelio Heredia-Jimenez, Roberto C. Ambrosio, Jesus Huerta-Chua, Hector De Cos-Cholula, Sergio Hernandez-Mendez

**Affiliations:** 1 Engineering School, University of Xalapa, Km. 2 Carretera Xalapa-Veracruz, Xalapa, Veracruz 91190, Mexico; guda.diaz.gd@gmail.com (G.D.-A.); sergio.h@ux.edu.mx (S.H.-M.); 2 Facultad de Instrumentacion Electronica, Universidad Veracruzana, Cto. Gonzalo Aguirre Beltran S/N, Xalapa, Veracruz 91000, Mexico; vicjimenez@uv.mx; 3 Consejo Veracruzano de Investigacion Cientifica y Desarrollo Tecnologico (COVEICYDET), Av. Rafael Murillo Vidal No. 1735, Cuauhtemoc, Xalapa, Veracruz 91069, Mexico; 4 Electronics Department, National Institute for Astrophysics, Optics and Electronics, Sta. María Tonantzintla, Puebla 72840, Mexico; luish@inaoep.mx (L.H.-M.); hdecos@inaoep.mx (H.D.C.-C.); 5 Electronics Department, UPAEP, 21 Sur 1103, Puebla 72410, Mexico; aureliohoracio.heredia@upaep.mx; 6 Faculty of Electronics Science Meritorious University Autonomous of Puebla, 4 Sur 104 Centro, Puebla 72000, Mexico; roberto.ambrosio@correo.buap.mx; 7 Instituto Tecnologico Superior de Poza Rica, Tecnologico Nacional de Mexico, Luis Donaldo Colosio Murrieta S/N, Arroyo del Maiz, Poza Rica, Veracruz 93230, Mexico; chua@itspozarica.edu.mx

**Keywords:** robot motion, path planning, piecewise linear approximation, multiple-target path planning, autonomous mobile robot, homotopy based path planning

## Abstract

The ability to plan a multiple-target path that goes through places considered important is desirable for autonomous mobile robots that perform tasks in industrial environments. This characteristic is necessary for inspection robots that monitor the critical conditions of sectors in thermal, nuclear, and hydropower plants. This ability is also useful for applications such as service at home, victim rescue, museum guidance, land mine detection, and so forth. Multiple-target collision-free path planning is a topic that has not been very studied because of the complexity that it implies. Usually, this issue is left in second place because, commonly, it is solved by segmentation using the point-to-point strategy. Nevertheless, this approach exhibits a poor performance, in terms of path length, due to unnecessary turnings and redundant segments present in the found path. In this paper, a multiple-target method based on homotopy continuation capable to calculate a collision-free path in a single execution for complex environments is presented. This method exhibits a better performance, both in speed and efficiency, and robustness compared to the original Homotopic Path Planning Method (HPPM). Among the new schemes that improve their performance are the Double Spherical Tracking (DST), the dummy obstacle scheme, and a systematic criterion to a selection of repulsion parameter. The case studies show its effectiveness to find a solution path for office-like environments in just a few milliseconds, even if they have narrow corridors and hundreds of obstacles. Additionally, a comparison between the proposed method and sampling-based planning algorithms (SBP) with the best performance is presented. Furthermore, the results of case studies show that the proposed method exhibits a better performance than SBP algorithms for execution time, memory, and in some cases path length metrics. Finally, to validate the feasibility of the paths calculated by the proposed planner; two simulations using the pure-pursuit controlled and differential drive robot model contained in the Robotics System Toolbox of MATLAB are presented.

## 1. Introduction

Autonomous mobile robot is an entity capable of performing a wide variety of tasks that involve displacement in its workspace, such as home service, pickup and delivery assistance in offices, monitoring factories, and so forth. An autonomous robot develops its assignment without human intervention because it commonly implies a certain degree of risk for a human being or environmental conditions make the use of teleoperation impossible. To execute its task, the robot must be capable to plan a path from an initial position to a target-position. In principle, path planning is a geometric process in which an autonomous agent must find a collision-free path in its workspace, without considering its kinematics and dynamics restrictions [1,2,3,4]. Then, once a path is specified, another process is executed to calculate the motion plan using the kinodynamic properties of the robot. Usually, for a path planning process, the robot is considered as a point in the configuration space ($C_{\mathrm{space}}$), which is the space generated by all feasible positions that it can reach [3,5,6]. Then, $C_{\mathrm{space}}$ is divided into free configuration space ($C_{\mathrm{free}}$) for valid positions and obstacles space ($C_{\mathrm{obs}}$) for all forbidden configurations.

### 1.1. Planning Algorithms

Finding a collision-free path in $C_{\mathrm{free}}$ may seem like an easy task for a human agent, nevertheless, it is a very complex issue for artificial intelligence. This has been the main motivation for researchers and developers community which have worked in this area during the last five decades [2,3,4,6,7,8,9,10,11]. These efforts have generated diverse planning algorithms with particular characteristics that define its degree of completeness, approach, and configuration of problems that each planner is capable of solve. In general, the planners can be classified into three categories.

1. Planners classified according to its completeness are: (I) Complete. These algorithms can find one solution, if it exists; otherwise, reports a failure. The most common algorithms in this category are visibility graph, Voronoi diagrams, Delaunay triangulations, among others graph based planners [12,13,14,15]. (II) Semi-complete. These algorithms can find a solution if one exists; otherwise, it may run forever while a stop criterion is not reached. The most relevant planners in this category are the method of artificial potential fields (APF) [6,16,17,18] and homotopic path planning method (HPPM) [10,19,20,21]. (III) Resolution complete. For any algorithm in this category; the completeness is strongly related to the resolution, size, and shape of the cells in the grid. Here, if a solution exists, any of these algorithms can obtain one; otherwise, terminates and reports that no solution exists for the specified resolution. All planners in this category are based on a cell decomposition which uses a search algorithm to find the collision-free path. The most used search algorithms are Dijkstra’s, A*, the local current comparison, and any of its variants [22,23,24,25,26,27,28]. (IV) Probabilistically complete. The degree of completeness for any algorithm in this category is considered probabilistic, because, if a solution exists, the probability tends to one hundred percent as long as the number of iterations of this process tends to infinity. The most effective algorithms, in this category, are the sampling-based planners (SBP).
2. Planners classified according to its formulation or approach are: graph-based, cells-decomposition, artificial potential fields, sampling-based, and homotopy continuation methods. (a) Graph-based contains algorithms for whose their roadmap is modeled by a graph and a search algorithm is employed to obtain the valid path. (b) Cells decomposition contains all discrete-space based algorithms, generally, these are the best choice to obtain a valid path for maze-type environments. Nevertheless, for non-structured environments, its performance depends on the resolution of the grid. (c) Artificial potential fields contains little variants of the original method, in this classification a random optimization or another technique is implemented to deal with the local minimum problem. (d) Sampling-based contains all probabilistic-complete algorithms such as: probabilistic road maps (PRM) [7,29], expansive spaces trees (EST) [30], rapidly-exploring random trees (RRT) [3,7,31], bidirectional RRT (Bi-RRT, also named RRT-Connect) [32], RRT combined with a shortest path approach A (RRT*) [7], kynodinamic motion planning by interior-exterior cells of exploration (KPIECE) [1], and collision checking efficient algorithms (LazyPRM and LazyRRT) [33,34] which have the common modules; uniform distribution samples generator, collision checker, local planner, and smoothing post-processing algorithm [2,3,35]. (e) Homotopy continuation based planners are a new category that contains some variants of the original method introduced in Reference [19]. These variants have been proposed to improve performance, minimize computation time, and obtain the shortest path (reported in References [10,20,36,37]).
3. Planners are classified according to their ability to reuse pre-processed data for solving problems into: (a) single-query and (b) multiple-query. In this way, the algorithms and methods mentioned previously, as well as their variants, fall in one of these categories. On the one hand, multiple-query algorithms are commonly applied to solve static environments and the roadmap generated can be reused as many times as needed. Therefore, queries are very fast, nevertheless, the computational cost and time to generate the roadmap are impractical for dynamic environments; algorithms like PRM and graph-based (Visibility graphs and A*) have this property. On the other hand, for single-query planners, roadmap generation and extend function are developed in parallel to reduce the high computational cost of analyzing the entire environment. This characteristic makes these algorithms faster, nevertheless, the resulting roadmap is only useful for the current query. Some algorithms contained in this category are RRT, EST, KPIECE, artificial potential fields method, HPPM, among others (see Table 1).

Table 1 shows the main characteristics of the most used collision-free path planners. The last row presents the advantages of HPPM (the method of interest in this work) which makes it one of the most versatile and reliable planners, based on its single-query execution, deterministic approach, and quasi-complete success. Furthermore, this table remarks the main issue, or bottleneck, found in each sampling-based algorithm, APF method, visibility graph approach, A* algorithm, and HPPM. On the one hand, for SBP algorithms, the bottleneck is the computing time spent during the collision query procedure which consumes about sixty percent of the total time [9]. On the other hand, the main feature of LazzyRRT and LazzyPRM over RRT and PRM is the lower time for the collision query. Nevertheless, for these planners, the number of samples (density of nodes) has a high impact on its success to find a feasible path. For A* algorithm, its success lies in the resolution of the discretized workspace (better resolution implies higher memory consumption and execution time). For AFP, the main issue is the local minimum, which confine the robot and prevents the method to continue. For the visibility graph approach, the bottleneck is the calculation time, which increases according to the number of obstacles. As for HPPM, the main issue lies in the selection of the repulsion parameter for obstacles. This parameter plays an important role in this method because it determines the length of the path and, in consequence, the execution time. Although, the repulsion parameter selection for HPPM is considered solved for particular applications in References [38,39] and References [20,21,36,37]. A generalized strategy, at this moment, it is currently considered an open problem. One of the main contributions in the present work is the implementation of a systematic criterion to select the repulsion parameter to generate an optimal path, in terms of length and execution time, for HPPM.

### 1.2. Multiple-Target Path Planning

**Figure 1:**
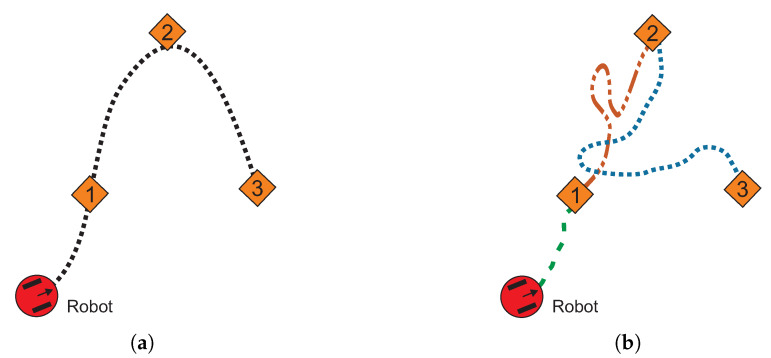
Efficient route and unnecessary turnings for multiple-target problem. (**a**) Efficient path for multiple-target problem. (**b**) Unnecessary turnings in multiple-target path (point-to-point strategy).

Commonly, the single-query and multiple-query collision-free planners are developed to find a valid path from a starting point to a single target point. Nevertheless, for applications like: robot vacuum cleaner, service at home, pickup and delivery services in office, industrial process and inspection, victim rescue, museum guidance, and land mine detectors, robots need to move through a sequence of target points [40,41,42,43,44,45]. The multiple-target collision-free path planning problem lacks deeper study, since it is considered a trivial task by using a point-to-point strategy. It operates through a set of sub-paths which creates a full multiple-target path, which connects each target point to another. While this strategy operates properly, its main disadvantage is the unnecessary and redundant turnings generated by connecting two or more segments; causing the sub-paths to cross each other and, in consequence, the robot will cross through the same place more than once [46] (see Figure 1b). In this sense, a complete path found through the point-to-point strategy is not optimal in terms of length.

**Figure 2:**
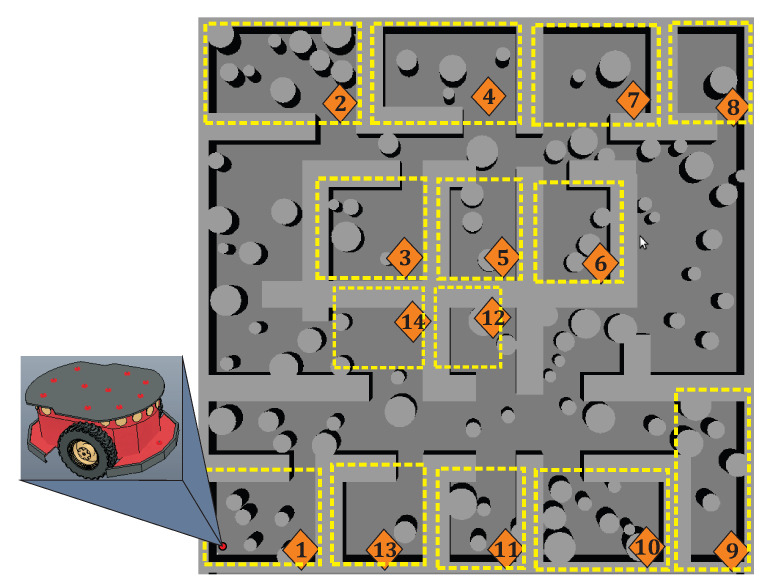
Multiple-target path planning issue in a complex environment (museum floor plan).

Figure 1a shows an example of desirable path (the shortest path) for a multiple-target problem. It can be noticed that the path is smooth without redundant configurations and pass very close to every target. On the other hand, Figure 1 depicts an example of point-to-point strategy for multiple-target problem. The unnecessary turnings and crossings between sub-paths are noticeable, making the trajectory inefficient in terms of distance. Figure 2 shows an example of a common office-like environment, this floor plan corresponds to a museum hall and the robot performs guidance tasks. In this case, it is considered that the robot calculates a path in some milliseconds, then, people and other dynamic agents could be represented as quasi-static obstacles (circular shapes in the environment map). The tasks of autonomous robots are to guide a group of people, provide relevant information about the displayed art, and visit all rooms while avoiding any collision with objects.

The museum environment in Figure 2 contains three common challenges for path planning algorithms. First, the map contains many obstacles that represent an issue for planners like BUG algorithm, artificial potential field method, and visibility graph methods. For these, the complexity and computing time increases accordingly to obstacles density. Second, the map displays several narrow corridors between walls and obstacles that are difficult to solve by SBP algorithms; while for artificial potential fields method this configuration exhibits local minimum problems. Third, for the layout in this map, the path should visit fourteen places, that is, the robot system must be capable to plan a path for multiple-target points. Although the first two issues have been thoroughly studied, multiple-targets is still considered as an open problem [40,44]. In this paper, a capable planner based on homotopy continuation to deal with narrow corridors, environment maps with hundreds of obstacles, and multiple-target issues is proposed. This paper is organized as follows. In Section 2, the bases of the homotopic path planning method are explained. The spherical algorithm applied to trace homotopy curves is presented in Section 3. Chua’s canonical piecewise linear model is shown in Section 4. Section 5 provides the main contributions and formulation of our proposed multiple-target HPPM. Furthermore, in Section 6, a HPPM with visibility approach is explained. Some case studies and performance comparison between the proposed methodology and SBP algorithms are presented in Section 7. Path tracking examples applying the pure-pursuit algorithm are provided in Section 8. Finally, the concluding remarks are given in Section 9.

## 2. Homotopic Path Planning Method

Homotopic path planning method (HPPM) is a single-query planner capable to obtain a collision-free path by using a mathematical model emanated from the configuration space, initial and final positions [10,19]. This planner is based on homotopy continuation methods (HCM) which, frequently, are employed to find multiple solutions of non-linear algebraic equation system (NAES) of the form

(1) $$f\left(X\right)=0:\;R^{n}⟶R^{n}.$$

The homotopy operates through a deformation of f(X) by adding a function G(X) and a homotopy parameter λ, such that

(2) H(X,λ)=λf(X)+(1−λ)G(X)=0,

where $H\left(f(X)\right)=H\left(X,\lambda \right):R^{n+1}⟶R^{n},\;X\in R^{n}\;,\;\lambda \in \left[0,1\right]$; G(X) is a function with a trivial or known solution. The homotopy system has the following properties

- For H(X,0)=0, the trivial or known solution is obtained.
- For H(X,1)=f(X), one solution of the original system is found.
- The homotopy curve is formed by the set of intersection points between the equations in the homotopy system (2), that is, $H\left(X,\lambda \right)=0:\left(X,\lambda \right)\in H^{-1}\left(0\right)$. Where, $H^{-1}\left(0\right)$ represents the set of intersections and is denoted, commonly, by γ [19,47,48,49]. Furthermore, all solutions of the original system f(X)=0 are included in $H^{-1}\left(0\right)$; these are found during the continuous deformation at λ=1.

HPPM process takes the system of equations that models the configuration space and, through the application of Newton’s homotopy, a free collision path is obtained. For Newton’s homotopy, the auxiliary function is $G\left(X\right)=f\left(X\right)-f\left(X_{0}\right)$; where $X_{0}$ is the known starting point. Then, HPPM employs the spherical algorithm (SA) to properly track the homotopy curve. For this system of equations, the curve represents a sequence of points that describes a continuous motion from a starting point to a target-point avoiding collisions with obstacles [10,19]. The configuration for a 2-D environment is presented as NAES $f_{1}\left(x,y\right)=0$ and $f_{2}\left(x,y\right)=0$. For both equations the unique solution lies on the target-point $\left(x_{T},y_{T}\right)$ [10,19]. The configuration space is modeled by the following equations

(3) $$f_{1}\left(x,y\right)=L_{1}\left(x,y\right)=0,$$

(4) $$f_{2}\left(x,y\right)=L_{2}\left(x,y\right)+g\left(x,y\right)=0,$$

where g(x,y) model the obstacles on the map, which creates singular regions around them. $L_{1}\left(x,y\right)$ and $L_{2}\left(x,y\right)$ are two auxiliary straight lines that intersect at the target-point, these are represented by

(5) $$L_{i}\left(x,y\right)=-y+m_{i}x+b_{i}=0;\;i=1,2,$$

then, applying Newton’s homotopy to Equation (3) and Equation (4), the NAES is transformed into

(6) $$H=\{\begin{array}{c} H_{1}\left(f_{1}\left(x,y\right),\lambda \right)=f_{1}\left(x,y\right)-\left(1-\lambda \right)f_{1}\left(x_{0},y_{0}\right)=0,\\ H_{2}\left(f_{2}\left(x,y\right),\lambda \right)=f_{2}\left(x,y\right)-\left(1-\lambda \right)f_{2}\left(x_{0},y_{0}\right)=0, \end{array}$$

where $\left(x_{0},y_{0}\right)$ is the initial point. The obstacles in the configuration space can be modeled by circumferences, ellipsoids, and other closed curves. For this work, only circular and quasi-rectangular shapes modeled using circle and ellipsoid equations [19], respectively are employed. Furthermore, the solid obstacles representation proposed in Reference [36] is used, such that each circular obstacle is defined by

(7) $$\left|C_{i}\left(x,y\right)\right|+C_{i}\left(x,y\right)=0,$$

where C(x,y) is the general equation of the circumference, modeled by

(8) $$C_{i}\left(x,y\right)={\left(x-x_{C,i}\right)}^{2}+{\left(y-y_{C,i}\right)}^{2}-r_{C,i}^{2}=0,$$

i=1,2,3,…,k, *k* is the number of circular obstacles in the map; $\left(x_{C,i},y_{C,i}\right)$ represents the center of the *i*-th circular obstacle, and $r_{C,i}$ its radius. The solid circle obstacle representation has solution for all points inside and at the contour of the circumference, that is, $\forall \;\left(x,y\right)\in R^{2}$ such that $\sqrt{{\left(x-x_{C,i}\right)}^{2}+{\left(y-y_{C,i}\right)}^{2}}\leq r_{C,i}$. Then, the expression representing all circular obstacles in the map is

(9) $$W_{C}\left(x,y\right)=\sum_{i=1}^{i=k}\frac{p_{C,i}}{C_{i}\left(x,y\right)+\left|C_{i}\left(x,y\right)\right|}\;,$$

here, $p_{C,i}$ is the repulsion parameter of the *i*-th circular obstacle [10,19]. Likewise, this principle is applied to ellipsoidal obstacles using

(10) $$R_{j}\left(x,y\right)={\left(\frac{x-x_{R,j}}{\alpha }\right)}^{2\eta }+{\left(\frac{y-y_{R,j}}{\beta }\right)}^{2\nu }-1=0,$$

where, the shape of each rectangular obstacle is defined by R(x,y) in Equation (10); j=1,2,3,…,l, *l* is the number of rectangular obstacles in the map; $\left(x_{R,j},y_{R,j}\right)$ is the center for the *j*-th rectangular obstacle; α and β are width and length, respectively; η and ν are two integers that define the sharp of the vertex [19,36]. The solid representation for ellipsoidal obstacles is modeled as

(11) $$\left|R_{j}\left(x,y\right)\right|+R_{j}\left(x,y\right)=0,$$

in a similar way to circular obstacles, this representation has a solution for all points inside and at the contour of the ellipsoid. Then, the expression that represents all ellipsoidal obstacles in the map is

(12) $$W_{R}\left(x,y\right)=\sum_{j=1}^{j=l}\frac{p_{R,j}}{R_{j}\left(x,y\right)+\left|R_{j}\left(x,y\right)\right|}\;,$$

$p_{R,j}$ is the repulsion parameter for the *j*-th ellipsoidal obstacle. It is important to notice that the points inside and at the contour of the obstacles result in divisions by zero for Equations (9) and (12). These divisions by zero creates singularities in the map, thus, HPPM uses them to automatically avoid collisions with the obstacles. The effect of all obstacles, whether they are ellipsoid or circular, are contained in the following expression

(13) $$W\left(x,y\right)=W_{C}\left(x,y\right)+W_{R}\left(x,y\right),$$

where $W_{C}\left(x,y\right)$ represents the solid circular obstacles and $W_{R}\left(x,y\right)$ represents the solid ellipsoidal obstacles. The term g(x,y) in Equation (4) is redefined as

(14) $$g\left(x,y\right)=W\left(x,y\right)-W\left(x_{T},y_{T}\right),$$

here, $W(x_{T},y_{T})$ is added to the NAES to cancel the effect of the obstacles at the target-point.

## 3. Homotopy Path Tracking Scheme

**Figure 3:**
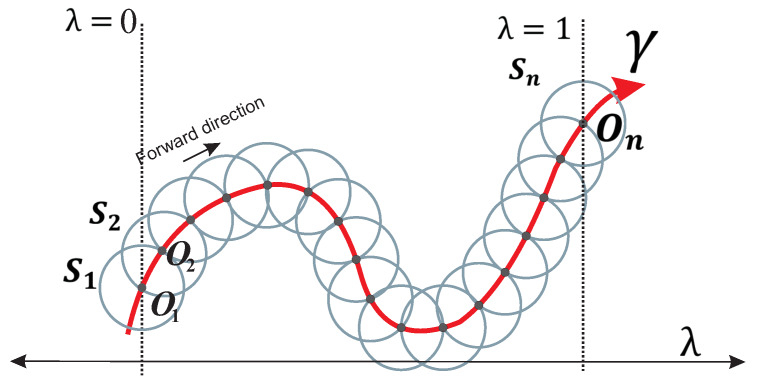
2-D representation of spherical tracking algorithm process.

Spherical algorithm (SA) is a tool applied to track continuous curves resulting from a non-homogeneous NAES. Previous works have demonstrated the effectiveness of SA to trace homotopy curves γ [10,36,47,48,49,50]. The homotopy is an operator such that $H\left(f(X)\right):R^{n+1}⟶R^{n}$, that is, homotopy system (6) is non-homogeneous. SA operates by adding an *n*-dimensional sphere equation $S_{i}\left(X,\lambda \right)$ (the dimension of sphere or hypersphere depends on the number of variables in the system of equations) that guarantees, at least, two cross points with the homotopy curve (6), as long as its center is at $H^{-1}\left(0\right)$ [47]. The homotopy process is described as follows: first step, the center of first sphere ($S_{1}$) is placed at $O_{1}=\left(x_{0},y_{0},\lambda =0\right)$ (see Figure 3). Then, the first root on the right is calculated by a predictor-corrector scheme; the root represents the intersection between the homotopy curve and the first hypersphere for values of λ≥0. The next step consists of the assignation of the calculated root on the right as the center ($O_{2}$) of a new hypersphere ($S_{2}$), as shown in Figure 3. Numerical path tracking scheme is based on

(15) $$H_{S}=\left\{\begin{array}{c} H_{1}\left(x,y,\lambda \right)=0,\\ H_{2}\left(x,y,\lambda \right)=0,\\ S_{i}\left(x,y,\lambda \right)=0, \end{array}\right.$$

for three dimensions (Bi-dimensional space and homotopy parameter λ), the sphere is represented as

(16) $$S_{i}\left(x,y,\lambda \right)={\left(x-c_{x}\right)}^{2}+{\left(y-c_{y}\right)}^{2}+{\left(\lambda -c_{\lambda }\right)}^{2}-r_{s}^{2}=0,$$

where $r_{s}$ is the radius and $O_{i}=\left(c_{x},c_{y},c_{\lambda }\right)$ is the center of the sphere for each step in the spherical tracking.

Homotopy curves tracking is a complex task, because the sharp turning points makes the convergence of the corrector step difficult [10,19,48,49]. In this regard, a proper predictor scheme is employed to improve the performance of the SA; in this work the Euler’s predictor is applied. Moreover, this scheme has shown adequate performance for tracking homotopy curves making the corrector scheme to converge faster [10,49]. This predictor scheme operates using the tangent vector to calculate an approximation of the next point in the tracking curve, as explained in the following section.

### 3.1. Euler’s Predictor Scheme

**Figure 4:**
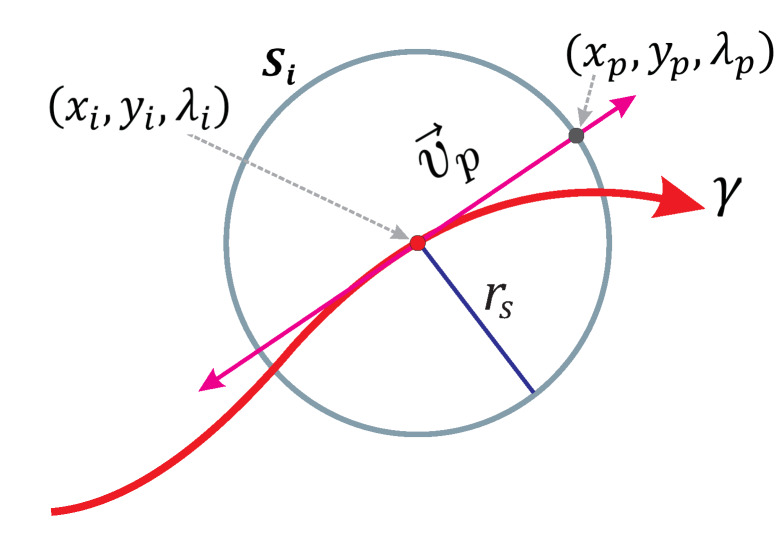
Euler’s predictor scheme.

Euler’s scheme is a complement of SA that provides a predictor point close to the intersection between homotopy curve and the respective sphere at each step of the tracking. Work in References [10,49] explain its operation and show its effectiveness as a predictor scheme in SA. For system equations with three variables, the Euler predictor point is calculated using

(17) $$\left(x_{p},y_{p},\lambda_{p}\right)=\left(x_{i},y_{i},\lambda_{i}\right)+r_{s}\left(\frac{{\vec{v}}_{p}}{∥{\vec{v}}_{p}∥}\right),$$

where $\left(x_{p},y_{p},\lambda_{p}\right)$ is the predictor point; $\left(x_{i},y_{i},\lambda_{i}\right)$ is the center of the sphere; $r_{s}$ is the radius of the sphere, and $\vec{v_{p}}$ is the tangent vector. Figure 4 shows a 2-D view of the predictor scheme operation in the SA. The predictor point $\left(x_{p},y_{p},\lambda_{p}\right)$ is located at the intersection between the vector point and the sphere $S_{i}$.

The tangent vector is obtained from the partial derivatives of the homotopy system (6), as explained in References [10,49]. This method uses partial derivatives with respect to an arbitrary parameter ρ, such that $\left(x(\rho ),y(\rho ),\lambda (\rho )\right)$; then, the chain rule is employed to calculate the tangent vector for the homotopy system at the point $\left(x_{i},y_{i},\lambda_{i}\right)$. Placing the tangent vector in Equation (17) the predictor point $\left(\left(x_{p},y_{p},\lambda_{p}\right)\right)$ is obtained

(18) $$\left(x_{p},y_{p},\lambda_{p}\right)=\left(x_{i},y_{i},\lambda_{i}\right)+r_{s}\frac{\left(x_{i}^{\prime }\left(\rho \right),y_{i}^{\prime }\left(\rho \right),\lambda_{i}^{\prime }\left(\rho \right)\right)}{∥(x_{i}^{\prime }\left(\rho \right),y_{i}^{\prime }\left(\rho \right),\lambda_{i}^{\prime }\left(\rho \right))∥}\;,$$

where,

(19) $$\left(x^{\prime }\left(\rho \right),y^{\prime }\left(\rho \right),\lambda^{\prime }\left(\rho \right)\right)=\left(\frac{\partial x_{i}\left(\rho \right)}{\partial \rho },\frac{\partial y_{i}\left(\rho \right)}{\partial \rho },\frac{\partial \lambda_{i}\left(\rho \right)}{\partial \rho }\right),$$

here, the partial derivative vector of each variable in the system with respect to ρ, evaluated at $\left(x_{i},y_{i},\lambda_{i}\right)$, represents the tangent vector [10,49].

### 3.2. Broyden’s Method as Corrector Scheme

The corrector scheme is the core of the HCM process, capable to obtain one solution for homogeneous NAES from an initial condition. Commonly, Newton-like correctors are described by the expression

(20) $$X_{i+1}=X_{i}-{\left[J(X_{i})\right]}^{-1}f\left(X_{i}\right),$$

here, $X\in R^{n}$, i=0,1,2,…,n; $X_{i+1}$ is the next point in the iterative process; $X_{i}$ is the current point and represents the initial condition for i=0, and ${\left[J(X_{i})\right]}^{-1}$ is the Jacobian inverse matrix of the NAES f(X). The iterative process ends when the stop criterion is achieved or the maximum number of iterations is reached; both parameters set by the user. Broyden’s method is a quasi-Newton method employed to calculate the roots of non-linear algebraic equation systems. For this, the Jacobian matrix $J(X_{i})$ is replaced by an approximation $A_{i}$ to reduce its calculation complexity. $A_{i}$ is calculated using

(21) $$A_{j}=A_{j-1}+\frac{f\left(X_{j}\right)-f\left(X_{j-1}\right)-A_{j-1}\left[X_{j}-X_{j-1}\right]}{∥X_{j}-X_{j-1}{∥}^{2}}{\left[X_{j}-X_{j-1}\right]}^{T},$$

for j=1,2,3,…,n; Broyden’s method is represented by

(22) $$X_{j+1}=X_{j}-{\left[A_{j}\left(X_{j}\right)\right]}^{-1}f\left(X_{j}\right),$$

where, in the first iteration (j=1), $X_{1}$ is calculated using Equation (20) and the matrix $A_{0}=J\left(X_{0}\right)$, that is, the Jacobian matrix only needs to be calculated once. Furthermore, the Jacobian matrix calculation is also employed to obtain the predictor point, hence, this matrix is strongly used in two complementary SA processes. In this work, Broyden’s Method stop criterion is set at i=20 or when the next criterion is fulfilled

(23) $$∥f\left(X_{i}\right)∥<1\times 10^{-9}.$$

**Figure 5:**
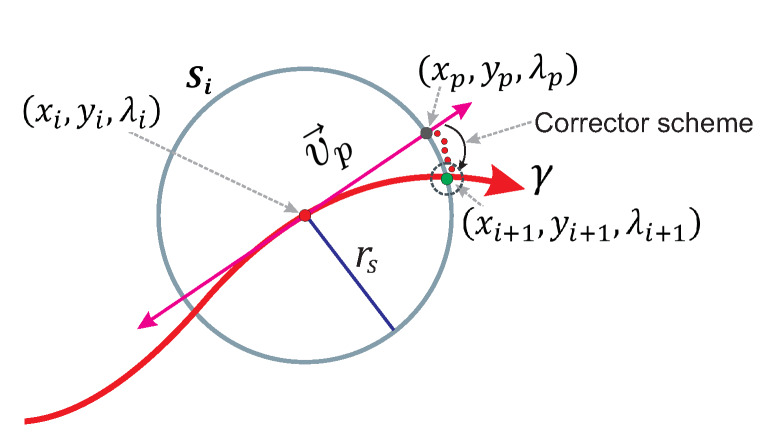
Corrector scheme for spherical tracking.

For the spherical algorithm, the initial point $X_{0}$ of Broyden’s method procedure is the predictor point $\left(x_{p},y_{p},\lambda_{p}\right)$ obtained using the Euler’s scheme. Then, the Broyden’s process is executed to solve the NAES system (15) for the current sphere ($S_{i}$) until the stop criterion is fulfilled. Which implies that the next solution point $\left(x_{i+1},y_{i+1},\lambda_{i+1}\right)$ has been found. Figure 5 depicts, in 2-D, the entire operation of the predictor-corrector scheme for SA.

## 4. Canonical Piecewise Linear Representation

A piecewise linear model can be defined as a mathematical representation that collects linear, related, descriptions to approximate a nonlinear function. The main reason that motivates the use of this type of model is the simplicity of their structure which allows an efficient implementation in algorithms. Although there are many piecewise linear models reported in the literature [51,52,53,54,55], due to its compact formulation, reduced number of parameters, and low computational requirements, the most popular is the so-called Chua’s model; it is described by a compact global representation named canonical piecewise linear function, given by

(24) $$y\left(x\right)=a+bx+\sum_{i=1}^{\sigma }c_{i}\left|x-x_{B,i}\right|=0,$$

where σ is the number of breakpoints. Model parameters *a*, *b*, and $c_{i}$, for i=1,2,...,σ, can be determined as follows

$$a=y\left(0\right)-\sum_{i=1}^{\sigma }c_{i}\left|x_{B,i}\right|,$$

$$b=\frac{J_{1}+J_{\sigma +1}}{2},$$

$$c_{i}=\frac{J_{i+1}-J_{i}}{2}.$$

**Figure 6:**
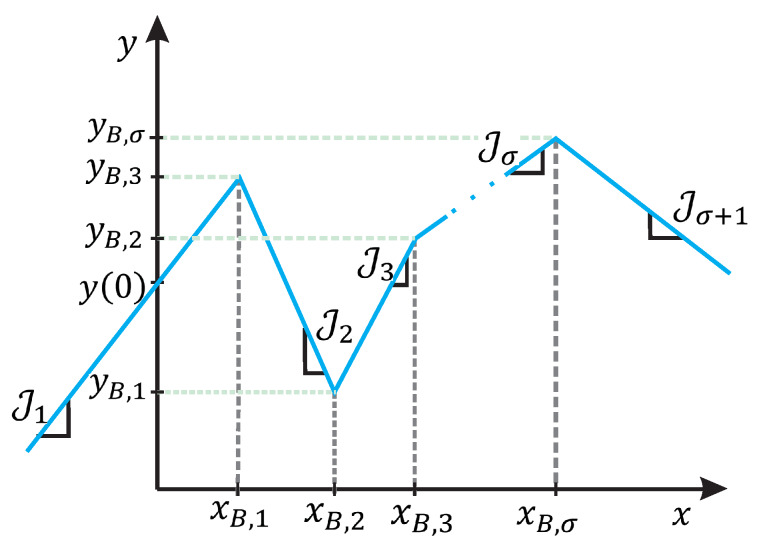
Single-valued piecewise linear function.

These parameters are strongly related to the graph of the piecewise linear function y(x). For instance, *b* and $c_{i}$ are both described in terms of $J_{i}$ which represents the slope of the *i*-th constitutive linear segment in the graph of y(x). Moreover, the parameter *a* is computed considering the σ-breakpoints $\left(x_{B,i},y_{B,i}\right)$, for i=1,2,...,σ included in the graph of y(x). To graphically illustrate this relation, Figure 6 shows a single-valued piecewise linear function constituted by σ breakpoints and σ+1 segments.

In this work, Chua’s model serves as the base for the proposed homotopy scheme and the two-dimensional PWL model is described by

(25) $$\mathrm{PWL}\left(x,y\right)=y-\left(a+bx+\sum_{i=1}^{\sigma }c_{i}\left|x-x_{B,i}\right|\right),$$

where PWL(x,y) is an implicit representation of Equation (24). It can notice, that depends on *x* values. The breakpoints $\left(x_{B,i},y_{B,i}\right)$ can take any value in the *y*-axis but, for *x*-axis, the values must be incremental such that $x_{B,i}<x_{B,i+1}<\ldots <x_{B,i+n}$.

## 5. Multiple-Target Homotopic Path Planning Method

This section is devoted to explain our proposed multiple-target homotopic path planning method (MTHPPM) and highlight the most relevant contributions of this work. MTHPPM is a planner based on the HPPM principle; for both, a set of auxiliary functions is necessary to generate a NAES in which the target-points are solutions. The multiple-target points in MTHPPM are provided by a set of two auxiliary functions that intersect in more of one point. In this work, target-points are generated by two single-valued PWL of the form Equation (24) with a similar formulation to the auxiliary functions in HPPM. These are represented by

(26) $$f_{{\mathrm{PWL}}_{1}}\left(x,y\right)={\mathrm{PWL}}_{1}\left(x,y\right),$$

(27) $$\begin{array}{cc} f_{{\mathrm{PWL}}_{2}}\left(x,y\right) & ={\mathrm{PWL}}_{2}\left(x,y\right)\\ \; & -g\left(x,y\right)(|{\mathrm{PWL}}_{2}\left(x,y\right)|+|{\mathrm{PWL}}_{1}\left(x,y\right)|), \end{array}$$

where g(x,y) is the expression that models all obstacles in the proposed MTHPPM, represented by

(28) $$g\left(x,y\right)=W_{C}\left(x,y\right)+W_{R}\left(x,y\right),$$

$W_{C}\left(x,y\right)$ and $W_{R}\left(x,y\right)$ are the mathematical representation of circular and ellipsoidal obstacles, modeled by Equation (12) and Equation (9), respectively. Then, the homotopy system (6) is redefined by Equation (32), Equation (33), and Newton’s homotopy formulation, obtaining

(29) $$H=\{\begin{array}{cc} H_{1}\left(f_{{\mathrm{PWL}}_{1}}\left(x,y\right),\lambda \right) & =f_{{\mathrm{PWL}}_{1}}\left(x,y\right)\\ \; & -\left(1-\lambda \right)f_{{\mathrm{PWL}}_{1}}\left(x_{0},y_{0}\right)=0,\\ H_{2}\left(f_{{\mathrm{PWL}}_{2}}\left(x,y\right),\lambda \right) & =f_{{\mathrm{PWL}}_{2}}\left(x,y\right)\\ \; & -\left(1-\lambda \right)f_{{\mathrm{PWL}}_{2}}\left(x_{0},y_{0}\right)=0, \end{array}$$

where $\left(x_{0},y_{0}\right)$ is the initial point of the trajectory.

In addition to the PWL auxiliary lines, the second expression (27) is reformulated to keep the properties of obstacles for the multiple-target points approach. This reformulation guarantees that, when ${\mathrm{PWL}}_{1}\left(x,y\right)=0$ and ${\mathrm{PWL}}_{2}\left(x,y\right)=0$, the effect of the obstacles function g(x,y) over the homotopy curve is cancelled. In other words, the effect of the obstacles over the homotopy system progressively vanishes as the robot approaches to any target point, that is, this effect decreases in proportion to the difference between ${\mathrm{PWL}}_{1}\left(x,y\right)$ and ${\mathrm{PWL}}_{2}\left(x,y\right)$. The absolute values are employed in this formulation because any change in the sign of g(x,y) modifies the behavior of the homotopy curve, as it is presented in References [10,20]. It can be noticed that absolute value terms produce a singular Jacobian matrix during the iterative process. Then, in order to solve these issues and improve the performance of MGHPPM, a differentiable approximation of absolute value is used.

### 5.1. Approximation of Absolute Value Function to Improve the Piecewise Linear Approach

**Figure 7:**
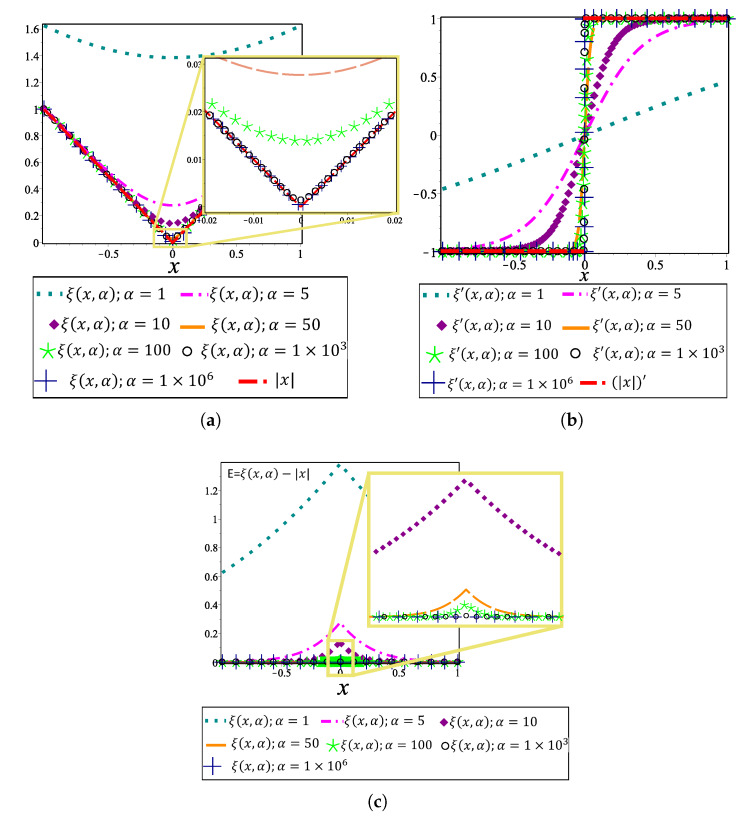
Approximation of absolute value function. (**a**) Approximation of absolute value function. (**b**) First derivation of the approximation of the absolute value function. (**c**) Error (E) between the approximation of absolute value function and |x|.

From definition of f(x)=|x|, f(x) is continuous for all values of *x*. Nevertheless, it is not differentiable when x=0. Then, the absolute value function is not differentiable at the breakpoints, that is, for all points (x,y) such that $x=x_{B,i}$ (see expression (24)). In order to remove this mathematical issue in the procedure, an approximation of the absolute value function is employed. For this work, the approximation of the absolute value function presented in Reference [56] is used to warrant the continuity of the PWL derivative function. This formulation is defined by

(30) $$\left|x\right|\approx \xi \left(x,\alpha \right)=\frac{1}{\alpha }\left(\ln\left(1+e^{-\alpha x}\right)+\ln\left(1+e^{\alpha x}\right)\right),$$

where α is a parameter which reduces the error between |x| and ξ(x). Figure 7a shows the approximation of ξ(x,α) to |x| when α→∞, this can also can be observed in Figure 7c. The first derivation graphs for ξ(x,α) and |x| are presented in Figure 7b. It shows that, like in the previous figure, the approximation ξ(x,α) is more similar to |x| when α→∞.

By using this approximation of absolute value function, the canonical PWL(x,y) representation is modified to generate smooth and differentiable auxiliary PWL functions ($\tilde{\mathrm{PWL}}\left(x,y\right)$) for MGHPPM. The $\tilde{\mathrm{PWL}}\left(x,y\right)$ representation is expressed as

(31) $$\tilde{\mathrm{PWL}}\left(x,y\right)=y-\left(a+bx+\sum_{i=1}^{i=\sigma }c_{i}\left(\xi (x-x_{B,i},\alpha )\right)\right),$$

**Figure 8:**
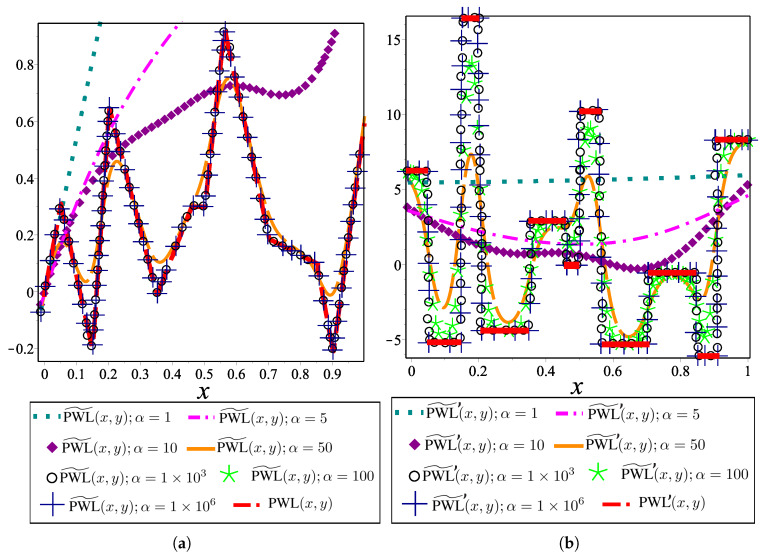
Smooth and differentiable piecewise linear function ($\tilde{\mathrm{PWL}}$). (**a**) $\tilde{\mathrm{PWL}}$ function generated with approximation of absolute value function. (**b**) First derivation of smooth $\tilde{\mathrm{PWL}}$ representation.

Figure 8a presents a $\tilde{\mathrm{PWL}}$ function generated with the canonical representation for $\alpha =1,5,10,50,1\times 10^{3},1\times 10^{9}$ in ξ(x,α). The first derivation of $\tilde{\mathrm{PWL}}\left(x,y\right)$ is depicted in Figure 8b which shows the discontinuities present in ${\mathrm{PWL}}^{\prime }\left(x,y\right)$. It is important to note that for values of α≤50, the behaviour of $\tilde{\mathrm{PWL}}\left(x,y\right)$ is unsuitable for the MTHPPM formulation due to the inaccuracy between PWL(x,y) and $\tilde{\mathrm{PWL}}\left(x,y\right)$ for these values of α, as it is depicted in Figure 8a,b. Nevertheless, a good approximation of PWL(x,y) is reached with $\alpha =1\times 10^{3}$ and $\alpha =1\times 10^{9}$ in $\tilde{\mathrm{PWL}}\left(x,y\right)$ with the advantage that these have continuous differentiation, as it is shown in Figure 8b.

For the MGHPPM, breakpoints are only used to generate intersections between auxiliary functions, in this sense, a good approximation that achieves this statement is when $\alpha =1\times 10^{3}$ (see Figure 8a). Then, this value is used for all case studies presented in the following section where, in order to simplify the notation, $\xi (x,1\times 10^{3})$ is denoted by ξ(x) and expresions (26), (27) and (29) are redefined as

(32) $$f_{{\tilde{\mathrm{PWL}}}_{1}}\left(x,y\right)={\tilde{\mathrm{PWL}}}_{1}\left(x,y\right),$$

(33) $$\begin{array}{ccc} f_{{\tilde{\mathrm{PWL}}}_{2}}\left(x,y\right) & = & {\tilde{\mathrm{PWL}}}_{2}\left(x,y\right)\\ \; & - & g\left(x,y\right)\left(\xi \left({\tilde{\mathrm{PWL}}}_{2}\left(x,y\right)\right)+\xi \left({\tilde{\mathrm{PWL}}}_{1}\left(x,y\right)\right)\right), \end{array}$$

(34) $$H=\{\begin{array}{cc} H_{1}\left(f_{{\tilde{\mathrm{PWL}}}_{1}}\left(x,y\right),\lambda \right) & =f_{{\tilde{\mathrm{PWL}}}_{1}}\left(x,y\right)\\ \; & -\left(1-\lambda \right)f_{{\tilde{\mathrm{PWL}}}_{1}}\left(x_{0},y_{0}\right)=0,\\ H_{2}\left(f_{{\tilde{\mathrm{PWL}}}_{2}}\left(x,y\right),\lambda \right) & =f_{{\tilde{\mathrm{PWL}}}_{2}}\left(x,y\right)\\ \; & -\left(1-\lambda \right)f_{{\tilde{\mathrm{PWL}}}_{2}}\left(x_{0},y_{0}\right)=0, \end{array}$$

### 5.2. Breakpoints Selection for the Piecewise-Linear Functions

**Figure 9:**
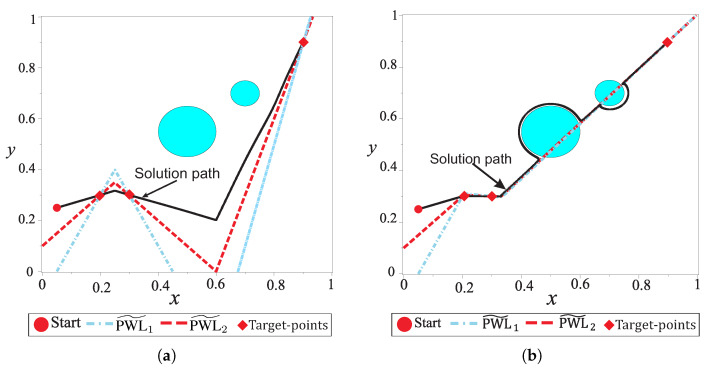
Selection of breakpoints for the $\tilde{\mathrm{PWL}}$ functions. (**a**) Breakpoints placed far from target-points. (**b**) Breakpoints placed close to target-points.

A set of proper auxiliary $\tilde{\mathrm{PWL}}$ functions generate the least number of unnecessary turns over the homotopy path. For this work, the way to obtain these are from two different initial points y(0) and the same breakpoints on the *x*-axis for both. This strategy guarantees that functions are different and intersect only at the desired points. Breakpoints are calculated from initial-points and target-points using the expressions

(35) $$x_{B,i}=x_{T,i}+\frac{x_{T,i+1}-x_{T,i}}{\rho },\;\rho =2,3,4,\ldots ,<\infty$$

(36) $$y_{B,i}=J_{i}\;x_{B,i}+y_{B,i-1}-J_{i}\left(x_{B,i-1}-x_{B,i}\right),$$

(37) $$J_{i}=\frac{y_{T,i}-y_{B,i-1}}{x_{T,i}-x_{B,i-1}},$$

where $\rho \in R^{+}$ is a proportionality parameter and determines the proximity between the *i*-th solution point and the *i*-th breakpoint (i=1,2,3,…,σ); for σ number of breakpoints there are σ+1 target-points; $\left(x_{B,i},y_{B,i}\right)$ is the position of the *i*-th breakpoint; $\left(x_{T,i},y_{T,i}\right)$ is the position of the *i*-th target-point; $J_{i}$ is the slope of the *i*-th segment of the $\tilde{\mathrm{PWL}}$ function, and $\left(x_{B,0}=0,y_{B,0}=y\left(0\right)\right)$ is the initial-point of the $\tilde{\mathrm{PWL}}$ function. A simple environment map with two circular obstacles and two variants of ${\tilde{\mathrm{PWL}}}_{1}$ and ${\tilde{\mathrm{PWL}}}_{2}$ configurations is shown in Figure 9. For the $\tilde{\mathrm{PWL}}$ functions in Figure 9a, the parameter is ρ=2; for Figure 9b, the parameter ρ=20 means that in this figure breakpoints are closer to the target-points.

Figure 9b shows the similarity between ${\tilde{\mathrm{PWL}}}_{1}$ and ${\tilde{\mathrm{PWL}}}_{2}$, nevertheless, these only intersect at target-points. Figure 9a,b show the effect of ${\tilde{\mathrm{PWL}}}_{1}$ and ${\tilde{\mathrm{PWL}}}_{1}$ in the homotopy formulation through Equation (33), where, the similarity of these in the homotopy formulation produces path with a similar tendency than $\tilde{\mathrm{PWL}}$’s. Figure 9b shows two $\tilde{\mathrm{PWL}}$’s which are overlapped to obstacles, this configuration produces that homotopy path pass very close to obstacles. This characteristic could be desirable in some cases, but when the map contains several grouped obstacles, the solution path could be inefficient in terms of length. In order to obtain paths very close to direct path between two points, for this work, the breakpoints are placed close to the target-points (similar form than Figure 9b).

### 5.3. Technique for Successful Convergence and Avoid Reversal Effect

**Figure 10:**
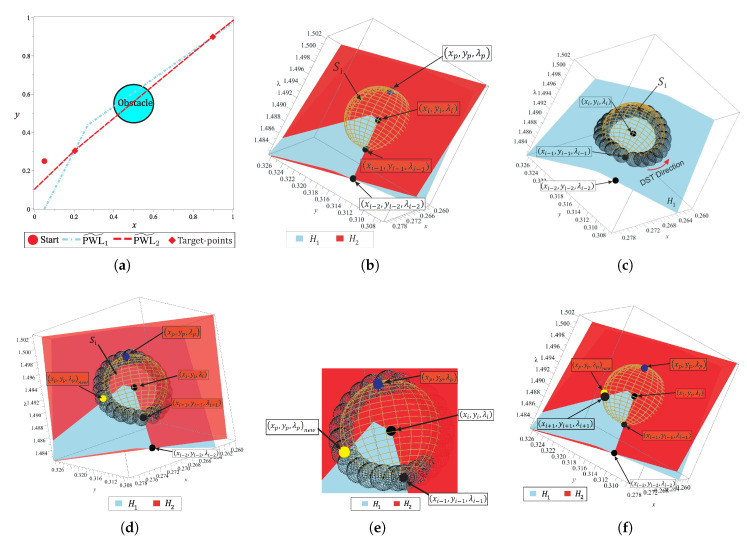
Double spherical tracking strategy. (**a**) Environment map with two target-points. (**b**) Non-convergence issue. (**c**) Double spherical tracking $H_{1}$ and $S_{i}$. (**d**) Double spherical tracking. (**e**) Double spherical tracking (zoom view). (**f**) New predictor-point.

Reversal effect is a phenomenon inherent to spherical path tracking and it is one of the most complex problems for the tracking techniques. Some works have proposed strategies to deal with this issue [10,47,48], nevertheless, these are inefficient when a $\tilde{\mathrm{PWL}}$ approach is attempted. This section introduces a new strategy to avoid reversal effect during the tracking and improve the convergence of the corrector scheme. The proposed strategy is named double spherical tracking (DST); it is a spherical tracking embedded into another spherical tracking. DST is executed when a reversal effect or a non-convergence is detected. On the one hand, the reversal effect is recognized using the directional cosine strategy as explained in References [10,48]. On the other hand, a non-convergence is detected when the maximum number of iterations is reached without meeting the stop criterion. DST procedure is explained using the non-convergence case shown in Figure 10. Figure 10a shows a simple map with one obstacle and two target-points. Figure 10b shows the position of the predictor point $\left(x_{p},y_{p},\lambda_{p}\right)$ which is far from the intersection between $H_{1}\left(x,y,\lambda \right)$, $H_{2}\left(x,y,\lambda \right)$, and $S_{i}\left(x,y,\lambda \right)$. DST formulation is based on the principle that $\forall {\left(x,y,\lambda \right)}_{sol}\in H_{S}^{-1}\left(0\right)\Rightarrow {\left(x,y,\lambda \right)}_{sol}\in H_{1,S}^{-1}\left(0\right)$. Where, $H_{s}^{-1}\left(0\right)$ is the solution set for the system of Equations (15) and $H_{1,S}^{-1}\left(0\right)$ is the solution set for the system of equations

(38) $$H_{1,S}=\left\{\begin{array}{c} H_{1}\left(x,y,\lambda \right)=0,\\ S_{i}\left(x,y,\lambda \right)=0, \end{array}\right.$$

The procedure steps of DST are described as follows:

1. The system of equations $H_{\mathrm{DST}}$ is established from $H_{1}\left(x,y,\lambda \right)$, $S_{i}\left(x,y,\lambda \right)$, and the sphere DST(x,y,λ). DST has been formulated to track the curve of intersection between $H_{1}\left(x,y,\lambda \right)$ and $S_{i}\left(x,y,\lambda \right)$ using the SA algorithm.
  (39) $$H_{\mathrm{DST}}=\left\{\begin{array}{c} H_{1}\left(x,y,\lambda \right)=0,\\ S_{i}\left(x,y,\lambda \right)=0,\\ {\mathrm{DST}}_{k}\left(x,y,\lambda \right)=0, \end{array}\right.$$
  (40) $$\begin{array}{c} {\mathrm{DST}}_{k}\left(x,y,\lambda \right)={\left(x-{\mathrm{dc}}_{x}\right)}^{2}+{\left(y-{\mathrm{dc}}_{y}\right)}^{2}\\ +{\left(\lambda -{\mathrm{dc}}_{\lambda }\right)}^{2}-r_{\mathrm{dst}}^{2}=0, \end{array}$$
  where k=1,2,3,…,n, *n* is the number of DST steps; $r_{\mathrm{dst}}$ is the radius, and $\left({\mathrm{dc}}_{x},{\mathrm{dc}}_{y},{\mathrm{dc}}_{\lambda }\right)$ is the center of the sphere for every *k*-th step of the DST.
2. The first sphere ${\mathrm{DST}}_{i}\left(x,y,\lambda \right)$ is placed at $O_{i-1}$ point, that is, ${\left({\mathrm{dc}}_{x},{\mathrm{dc}}_{y},{\mathrm{dc}}_{\lambda }\right)}_{1}=\left(x_{i-1},y_{i-1},\lambda_{i-1}\right)$. Figure 10b indicates the position of point $\left(x_{i-1},y_{i-1},\lambda_{i-1}\right)$, notice that it is at the intersection between all members of the system of Equation (15).
3. The SA algorithm is executed using the predictor and corrector schemes, explained above, for the non-linear system of Equation (39). Figure 10c depicts the schematic operation of DST; it can be noticed that the procedure starts at $\left(x_{i-1},y_{i-1},\lambda_{i-1}\right)$ point and continues until its initial point is reached, it means that DST tracks a closed curve. It is important to note that the system of Equation (39) are easier to track with less computation cost than the system of Equation (15). For his paper, the DST stop criterion is based on the distance between initial point of DST procedure $\left(x_{i-1},y_{i-1},\lambda_{i-1}\right)$ and the current DST solution ${\left({\mathrm{dc}}_{x},{\mathrm{dc}}_{y},{\mathrm{dc}}_{\lambda }\right)}_{k}$ as
  (41) $$∥{\left({\mathrm{dc}}_{x},{\mathrm{dc}}_{y},{\mathrm{dc}}_{\lambda }\right)}_{k}-\left(x_{i-1},y_{i-1},\lambda_{i-1}\right)∥<r_{\mathrm{dst}}$$
  here, the radius of DST sphere is proposed as $r_{\mathrm{dst}}=\left(2\pi r_{s}\right)/n_{\mathrm{dst}}$, where $r_{s}$ is the radius of the sphere $S_{i}$ of the spherical tracking. $n_{\mathrm{dst}}$ is the minimum number of steps of DST which is set to 24.
4. Finally, all points ${\left({\mathrm{dc}}_{x},{\mathrm{dc}}_{y},{\mathrm{dc}}_{\lambda }\right)}_{k}$, except $({\mathrm{dc}}_{x},{\mathrm{dc}}_{y},$${\mathrm{dc}}_{\lambda }{)}_{1}$, are evaluated in Equation (15). Then, the point for which the evaluation is closer to zero, $H_{S}\left({\left({\mathrm{dc}}_{x},{\mathrm{dc}}_{y},{\mathrm{dc}}_{\lambda }\right)}_{k}\right)\approx 0$, is taken as a new predictor in the SA tracking of $H_{S}$. Figure 10d shows the new predictor point ${\left(x_{p},y_{p},\lambda_{p}\right)}_{\mathrm{new}}$ which is a point from the solution set $H_{\mathrm{DST}}^{-1}\left(0\right)$. Figure 10e provides a closer view where ${\left(x_{p},y_{p},\lambda_{p}\right)}_{\mathrm{new}}$ is close to the intersection between $H_{1}\left(x,y,\lambda \right)$, $H_{2}\left(x,y,\lambda \right)$ and $S_{i}\left(x,y,\lambda \right)$. Then, this point is taken as the predictor point in the Broyden’s corrector scheme. The position of the new point $\left(x_{i+1},y_{i+1},\lambda_{i+1}\right)$ can be seen in Figure 10f.

**Figure 11:**
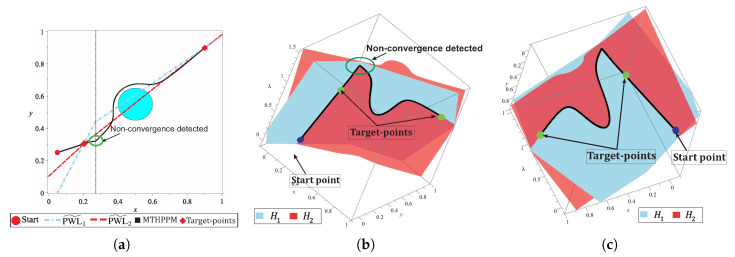
2-D and 3-D representation of the solution path. (**a**) Solution path 2-D representation. (**b**) Solution path 3-D representation (View 1). (**c**) Solution path 3-D representation (View 2).

It is important to remark that, DST is a backup technique for SA which is only applied when a non-convergence or reversal phenomenon is detected. Furthermore, the execution time of this technique is comparable with the execution time spent by one step of the SA. This because the $H_{\mathrm{DST}}$ system of equations does not contain the obstacles representation in its formulation. For the map shown in Figure 10a, the DST strategy is employed only once for the non-convergence point found during the tracking. Figure 11 shows the 2-D and 3-D representation of the NAES and homotopy surfaces, the region where is located the non-convergence point is indicated.

Figure 11a shows the successful solution path obtained once that non-convergence is solved. Figure 11b,c show two different 3-D views of the surfaces which correspond at each equation of $H_{S}$. In these figures, it can be noticed that the solution path represents the intersection between $H_{1}\left(x,y,\lambda \right)$ and $H_{2}\left(x,y,\lambda \right)$, crossing the two target-points. In this section, the DST technique for solving a non-convergence is explained and its ability to solve the reversion phenomenon is demonstrated.

### 5.4. A Dummy Obstacle to Improve the Spherical Algorithm Performance

**Figure 12:**
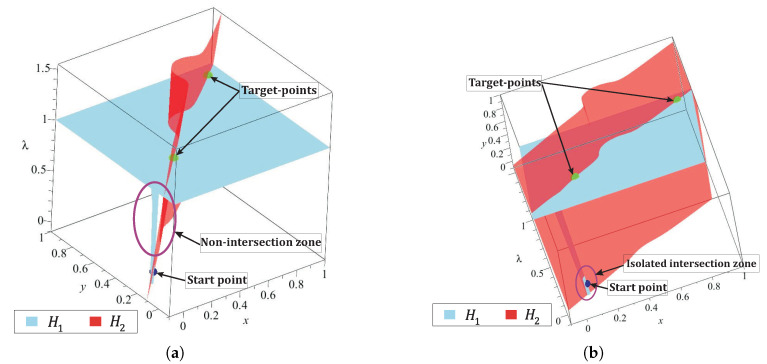
Map containing a dummy obstacle with isolated intersection zone. (**a**) Homotopy surfaces representation (View 1). (**b**)Homotopy surfaces representation (View 2).

Spherical tracking is an iterative process in which execution time and success depends on three main factors: predictor-corrector scheme, the radius of the sphere, and length of the path. First, the predictor-corrector scheme defines the total execution time, while the radius of the sphere and length of the path determines the number of predictor-corrector executions. For each SA step, the predictor is only employed one time, meanwhile, the number of iterations in Broyden’s corrector depends on the predictor point. In other words, the success and fast convergence of the Broyden’s corrector scheme depends on the quality of the predictor point. For quality, it means that proximity of the predictor point to the intersection between homotopy curve and the *i*-th sphere intersection (SA solution point). Then, the time for tracking an entire homotopy curve, from initial-point to target-point, is mostly determined by the total number of corrector scheme executions. In addition, the execution time for each corrector scheme iteration is the same for all SA steps; this implies a linear relationship between the total number of corrector iterations and the total computing time, as explained in Reference [10]. Second, if the radius of the sphere is reduced, the SA needs more steps to cover the same distance that could be traced by bigger spheres. Nonetheless, the sphere radius is restricted by the size of the obstacles, that is, if the sphere is bigger than the obstacles the SA could lose the path [10]. Third, the number of corrector executions increases as the homotopy curve length increases. The increase in length is due to the homotopy system formulation, as explained in Reference [10]. For homotopy with $\tilde{\mathrm{PWL}}$ formulation, the length of the curve drastically increases caused by turnings when slopes changes. For the implementation in this work, the homotopy curves generated by $\tilde{\mathrm{PWL}}$ formulation (34) are mapped in the space x∈(0,1), y∈(0,1) and λ∈(−∞,+∞). The plane x−y is delimited by the normalization of the workspace but λ is unlimited, nevertheless, for homotopy methods, the region of interest is λ∈(0,1). Figure 11b,c show that displacement of the homotopy path ever the λ-axis is greater than over the x−y plane. Then, in order to minimize the λ-axis variations, a small perturbation in $H_{1}\left(x,y,\lambda \right)$ is proposed to generate a significant modification in the surface $H_{1}$. This perturbation is performed by $C_{d}$ which is a dummy obstacle placed very close to the initial point $\left(x_{0},y_{0}\right)$ with a radius smaller than the spheres employed in the SA. This obstacle generates a change of orientation of surface $H_{1}\left(x,y,\lambda \right)$, and their effect on the homotopy system is negligible for points far away from $\left(x_{0},y_{0}\right)$. By using the dummy obstacle, the surface $H_{1}\left(x,y,\lambda \right)$ is forced to change its orientation. This change relates to the nature of Newton’s homotopy which implies two properties in the new formulation: (a) its representation of the obstacles in the 3-D space are cylinders and rectangular prisms projections which are parallel to λ-axis. This can be concluded from the projections of Equations (7) and (10) expressions from 2-D to 3-D. Then, the new function that includes the dummy obstacle is represented by

(42) $$\begin{array}{c} f_{{\tilde{\mathrm{PWL}}}_{1}}\left(x,y\right)={\tilde{\mathrm{PWL}}}_{1}\left(x,y\right)\\ -\left(\frac{p_{C,d}}{C_{d}}\right)\left(\xi \left({\tilde{\mathrm{PWL}}}_{2}\left(x,y\right)\right)+\xi \left({\tilde{\mathrm{PWL}}}_{1}\left(x,y\right)\right)\right), \end{array}$$

where, $p_{C,d}$ is the repulsion parameter of $C_{d}$. For this work, $p_{C,d}=0.1$, this value guarantees a minimal effect of the dummy obstacle in regions close to the starting point. b) The dummy obstacle integration in the homotopy system implies that ${\tilde{\mathrm{PWL}}}_{2}\left(x,y\right)$ must be modified to guarantee a continuous homotopy curve from initial-point to target-points. Figure 12 shows the effect of the $C_{d}$ on the surface $H_{1}\left(x,y,\lambda \right)$. This surface is flattened and their variations over λ-axis is small for points away from the dummy obstacle. The use of a dummy obstacle allowed to reduce, substantially, the sweep on λ-axis for the homotopy system in the region away from this obstacle.

The dummy obstacle represents a singularity in the flat surface, then for points closer to it, the variations over λ-axis are noticeable as shown in Figure 12a. It can observe that a non-intersection zone created an inclination on surface $H_{2}\left(x,y,\lambda \right)$, caused by its $\tilde{\mathrm{PWL}}$ formulation. Figure 12b shows the isolated zone in which the spherical tracking should be confined. From the previous analysis, a solution for the isolated region is proposed and performed by the formulation of $C_{d}$

(43) $$C_{d}={\left(x-x_{C,d}\right)}^{2}+{\left(y-y_{C,d}\right)}^{2}-{\left(r_{C,d}\right)}^{2},$$

$$\begin{array}{cc} & x_{C,d}=x_{0}-r_{s},\\ & y_{C,d}=y_{0}-J_{1}\left(x_{0}-x_{C,d}\right),\\ & J_{1}=\frac{y_{G,1}-y_{0}}{x_{G,1}-x_{0}}, \end{array}$$

here, the radius of dummy obstacle $r_{C,d}=\frac{r_{s}}{100}$ is small enough to be disregarded by the spherical tracking; $\left(x_{0},y_{0}\right)$ is the start point, and $\left(x_{G,1},y_{G,1}\right)$ is the first target-point. From the previous analysis, the initial point $\left(x_{B,0},y_{B,0}\right)$ of ${\tilde{\mathrm{PWL}}}_{2}\left(x,y\right)$ which guarantees a continuous solution path from start to every target-points is calculated using the next expression

(44) $$\left(x_{B,0},y_{B,0}\right)=\left(0,y_{0}-J_{1}\;x_{0}\right),$$

**Figure 13:**
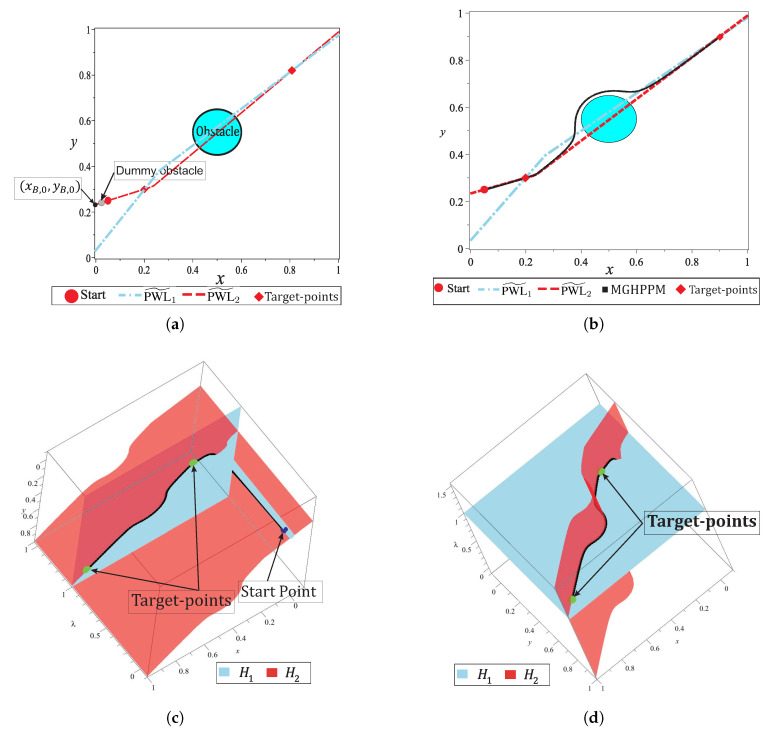
Map with added dummy obstacle. (**a**) Environment map with dummy obstacle and two target-points. (**b**) 2-D representation solution path. (**c**) [3-D representation (View 1) solution path. (**d**) 3-D representation (View 2) solution path.

Figure 13a depicts the dummy obstacle with center in $\left(x_{C,d},y_{C,d}\right)$ and using Equation (44) the point $\left(x_{B,0},y_{B,0}\right)$ for the ${\tilde{\mathrm{PWL}}}_{2}\left(x,y\right)$ is calculated. The successful solution path for multiple-target problem is shown in Figure 13b. Figure 13c,d show two views of the homotopy surfaces, $H_{1}\left(x,y,\lambda \right)$ and $H_{2}\left(x,y,\lambda \right)$. In these, it can notice the successful path begins at the start point and visits the two projected target-points. Figure 13d shows the effect of the dummy obstacle and the point $\left(x_{B,0},y_{B,0}\right)$ of ${\tilde{\mathrm{PWL}}}_{2}\left(x,y\right)$ to improve the homotopy path (in terms of length and number of SA stems). Furthermore, in this figure, the continuous curve denoted by the intersection between the homotopy surfaces $H_{1}$ and $H_{2}$ from the start point through all target-points is showed. The improvement of path (in terms of length) by using the dummy obstacle strategy can be validated from the visual comparison between the paths in the Figure 11 and Figure 13.

### 5.5. Strategy to Simplified the Jacobian Matrix Based on Symbolic Manipulation

To reduce the computational complexity and provide a simple implementation in C++ programming language, without the use of scientific specialized libraries, a symbolic manipulation of the Jacobian matrix (45) is developed. Jacobian matrix is the highest cost processes embedded in the SA; it is employed by predictor and corrector schemes for each step of the tracking. Furthermore, some terms also are employed on every step of DST.

(45) $$J\left(x,y,\lambda \right)=\left(\begin{array}{ccc} \frac{\partial H_{1}\left(x,y,\lambda \right)}{\partial x} & \frac{\partial H_{1}\left(x,y,\lambda \right)}{\partial y} & \frac{\partial H_{1}\left(x,y,\lambda \right)}{\partial \lambda }\\ \frac{\partial H_{2}\left(x,y,\lambda \right)}{\partial x} & \frac{\partial H_{2}\left(x,y,\lambda \right)}{\partial y} & \frac{\partial H_{2}\left(x,y,\lambda \right)}{\partial \lambda }\\ \frac{\partial S_{i}\left(x,y,\lambda \right)}{\partial x} & \frac{\partial S_{i}\left(x,y,\lambda \right)}{\partial y} & \frac{\partial S_{i}\left(x,y,\lambda \right)}{\partial \lambda } \end{array}\right)$$

The terms corresponding to the sphere $S_{i}$ and the partial derivatives to λ for $H_{1}$ and $H_{2}$ in the Jacobian matrix can be calculated by

1. From Equations (34) and (42)
  (46) $$\frac{\partial H_{1}\left(x,y,\lambda \right)}{\partial \lambda }={\tilde{\mathrm{PWL}}}_{1}\left(x_{0},y_{0}\right)-\left(\frac{p_{C,d}}{C_{d}}\right)\left(\xi \left({\tilde{\mathrm{PWL}}}_{2}\left(x_{0},y_{0}\right)\right)+\xi \left({\tilde{\mathrm{PWL}}}_{1}\left(x_{0},y_{0}\right)\right)\right),$$
2. From Equations (34) and (33)
  (47) $$\frac{\partial H_{2}\left(x,y,\lambda \right)}{\partial \lambda }={\tilde{\mathrm{PWL}}}_{2}\left(x_{0},y_{0}\right)-g\left(x_{0},y_{0}\right)\left(\xi \left({\tilde{\mathrm{PWL}}}_{2}\left(x_{0},y_{0}\right)\right)+\xi \left({\tilde{\mathrm{PWL}}}_{1}\left(x_{0},y_{0}\right)\right)\right),$$
3. From Equation (16)
  (48) $$\frac{\partial S_{i}\left(x,y,\lambda \right)}{\partial x}=2\left(x-c_{x}\right),$$
  (49) $$\frac{\partial S_{i}\left(x,y,\lambda \right)}{\partial y}=2\left(y-c_{y}\right),$$
  (50) $$\frac{\partial S_{i}\left(x,y,\lambda \right)}{\partial \lambda }=2\left(\lambda -c_{\lambda }\right),$$

Using symbolic manipulation and the chain rule to obtain the derivative of composite functions, and knowing that evaluation of $f_{{\tilde{\mathrm{PWL}}}_{1}}\left(x_{0},y_{0}\right)$ and $f_{{\tilde{\mathrm{PWL}}}_{2}}\left(x_{0},y_{0}\right)$ are constants, the rest of terms can be reduced as

1. From Equations (34) and (42)
  (51) $$\begin{array}{cc} \frac{\partial \left(H_{1}\left(x,y,\lambda \right)\right)}{\partial x}= & \frac{\partial \left({\tilde{\mathrm{PWL}}}_{1}\left(x,y\right)\right)}{\partial x}-\frac{\partial \left(\frac{p_{C,d}}{C_{d}\left(x,y\right)}\right)}{\partial x}\left(\xi \left({\tilde{\mathrm{PWL}}}_{2}\left(x,y\right)\right)+\xi \left({\tilde{\mathrm{PWL}}}_{1}\left(x,y\right)\right)\right)\\ \; & -\frac{\partial \left(\xi \left({\tilde{\mathrm{PWL}}}_{2}\left(x,y\right)\right)+\xi \left({\tilde{\mathrm{PWL}}}_{1}\left(x,y\right)\right)\right)}{\partial x}\left(\frac{p_{C,d}}{C_{d}\left(x,y\right)}\right), \end{array}$$
  (52) $$\begin{array}{cc} \frac{\partial \left(H_{1}\left(x,y,\lambda \right)\right)}{\partial y}= & \frac{\partial \left({\tilde{\mathrm{PWL}}}_{1}\left(x,y\right)\right)}{\partial y}\\ \; & -\frac{\partial \left(\frac{p_{C,d}}{C_{d}\left(x,y\right)}\right)}{\partial y}\left(\xi \left({\tilde{\mathrm{PWL}}}_{2}\left(x,y\right)\right)+\xi \left({\tilde{\mathrm{PWL}}}_{1}\left(x,y\right)\right)\right)\\ \; & -\frac{\partial \left(\xi \left({\tilde{\mathrm{PWL}}}_{2}\left(x,y\right)\right)+\xi \left({\tilde{\mathrm{PWL}}}_{1}\left(x,y\right)\right)\right)}{\partial y}\left(\frac{p_{C,d}}{C_{d}\left(x,y\right)}\right), \end{array}$$
2. From Equations (34) and (33)
  (53) $$\begin{array}{cc} \frac{\partial \left(H_{2}\left(x,y,\lambda \right)\right)}{\partial x}= & \frac{\partial \left({\tilde{\mathrm{PWL}}}_{2}\left(x,y\right)\right)}{\partial x}-\frac{\partial \left(g(x,y)\right)}{\partial x}\left(\xi \left({\tilde{\mathrm{PWL}}}_{2}\left(x,y\right)\right)+\xi \left({\tilde{\mathrm{PWL}}}_{1}\left(x,y\right)\right)\right)\\ \; & \frac{\partial \left(\xi \left({\tilde{\mathrm{PWL}}}_{2}\left(x,y\right)\right)+\xi \left({\tilde{\mathrm{PWL}}}_{1}\left(x,y\right)\right)\right)}{\partial x}\left(g(x,y)\right), \end{array}$$
  (54) $$\begin{array}{cc} \frac{\partial \left(H_{2}\left(x,y,\lambda \right)\right)}{\partial y}= & \frac{\partial \left({\tilde{\mathrm{PWL}}}_{2}\left(x,y\right)\right)}{\partial y}-\frac{\partial \left(g(x,y)\right)}{\partial y}\left(\xi \left({\tilde{\mathrm{PWL}}}_{2}\left(x,y\right)\right)+\xi \left({\tilde{\mathrm{PWL}}}_{1}\left(x,y\right)\right)\right)\\ \; & -\frac{\partial \left(\xi \left({\tilde{\mathrm{PWL}}}_{2}\left(x,y\right)\right)+\xi \left({\tilde{\mathrm{PWL}}}_{1}\left(x,y\right)\right)\right)}{\partial y}\left(g(x,y)\right), \end{array}$$
  where $\frac{\partial \left(g(x,y)\right)}{\partial x}$ and $\frac{\partial \left(g(x,y)\right)}{\partial y}$ are
  (55) $$\frac{\partial \left(g(x,y)\right)}{\partial x}=\sum_{i=1}^{i=k}\frac{-p_{O,i}\left(\frac{\partial \left(O_{i}\left(x,y\right)\right)}{\partial x}\right)}{{\left(\left|O_{i}\left(x,y\right)\right|+O_{i}\left(x,y\right)\right)}^{2}}\left(1+\frac{\left|O_{i}\left(x,y\right)\right|}{O_{i}\left(x,y\right)}\right),$$
  (56) $$\frac{\partial \left(g(x,y)\right)}{\partial y}=\sum_{i=1}^{i=k}\frac{-p_{O,i}\left(\frac{\partial \left(O_{i}\left(x,y\right)\right)}{\partial y}\right)}{{\left(\left|O_{i}\left(x,y\right)\right|+O_{i}\left(x,y\right)\right)}^{2}}\left(1+\frac{\left|O_{i}\left(x,y\right)\right|}{O_{i}\left(x,y\right)}\right),$$
  where $O_{i}\left(x,y\right)$ is the expression that describes the shape of the *i*-th obstacle; $C_{i}$ for a circular obstacle, and $R_{i}$ for an ellipsoidal obstacle. The parameter $p_{O,i}$ is the repulsion parameter of each obstacle; $p_{C,i}$ for a circular obstacle, and $p_{R,i}$ for an ellipsoidal obstacle.
  (57) $$\begin{array}{cc} \frac{\partial \left(\xi \left({\tilde{\mathrm{PWL}}}_{2}\left(x,y\right)\right)+\xi \left({\tilde{\mathrm{PWL}}}_{1}\left(x,y\right)\right)\right)}{\partial x} & =\frac{1}{\alpha }\frac{\partial \left({\tilde{\mathrm{PWL}}}_{2}\left(x,y\right)\right)}{\partial x}\left(-\frac{{\alpha_{e}}^{-\alpha {\tilde{\mathrm{PWL}}}_{2}\left(x,y\right)}}{1+e^{-\alpha {\tilde{\mathrm{PWL}}}_{2}\left(x,y\right)}}+\frac{{\alpha_{e}}^{\alpha {\tilde{\mathrm{PWL}}}_{2}\left(x,y\right)}}{1+e^{\alpha {\tilde{\mathrm{PWL}}}_{2}\left(x,y\right)}}\right)\\ \; & +\frac{1}{\alpha }\frac{\partial \left({\tilde{\mathrm{PWL}}}_{1}\left(x,y\right)\right)}{\partial x}\;\left(-\frac{{\alpha_{e}}^{-\alpha {\tilde{\mathrm{PWL}}}_{1}\left(x,y\right)}}{1+e^{-\alpha {\tilde{\mathrm{PWL}}}_{1}\left(x,y\right)}}+\frac{{\alpha_{e}}^{\alpha {\tilde{\mathrm{PWL}}}_{1}\left(x,y\right)}}{1+e^{\alpha {\tilde{\mathrm{PWL}}}_{1}\left(x,y\right)}}\right), \end{array}$$
  (58) $$\begin{array}{cc} \frac{\partial \left(\xi \left({\tilde{\mathrm{PWL}}}_{2}\left(x,y\right)\right)+\xi \left({\tilde{\mathrm{PWL}}}_{1}\left(x,y\right)\right)\right)}{\partial y} & =\frac{1}{\alpha }\frac{\partial \left({\tilde{\mathrm{PWL}}}_{2}\left(x,y\right)\right)}{\partial y}\;\left(-\frac{{\alpha_{e}}^{-\alpha {\tilde{\mathrm{PWL}}}_{2}\left(x,y\right)}}{1+e^{-\alpha {\tilde{\mathrm{PWL}}}_{2}\left(x,y\right)}}+\frac{{\alpha_{e}}^{\alpha {\tilde{\mathrm{PWL}}}_{2}\left(x,y\right)}}{1+e^{\alpha {\tilde{\mathrm{PWL}}}_{2}\left(x,y\right)}}\right)\\ \; & +\frac{1}{\alpha }\frac{\partial \left({\tilde{\mathrm{PWL}}}_{1}\left(x,y\right)\right)}{\partial y}\;\left(-\frac{{\alpha_{e}}^{-\alpha {\tilde{\mathrm{PWL}}}_{1}\left(x,y\right)}}{1+e^{-\alpha {\tilde{\mathrm{PWL}}}_{1}\left(x,y\right)}}+\frac{{\alpha_{e}}^{\alpha {\tilde{\mathrm{PWL}}}_{1}\left(x,y\right)}}{1+e^{\alpha {\tilde{\mathrm{PWL}}}_{1}\left(x,y\right)}}\right), \end{array}$$
  where, $\alpha =1\times 10^{3}$. Finally,
  (59) $$\frac{\partial \left({\tilde{\mathrm{PWL}}}_{1}\left(x,y\right)\right)}{\partial x}=-\left(b_{1}x+\sum_{i_{1}=1}^{i_{1}=\sigma }c_{i_{1}}\left(\frac{\xi \left(x-x_{B,i_{1}}\right)}{x-x_{B,i_{1}}}\right)\right),$$
  (60) $$\frac{\partial \left({\tilde{\mathrm{PWL}}}_{2}\left(x,y\right)\right)}{\partial x}=-\left(b_{2}x+\sum_{i_{2}=1}^{i_{2}=\sigma }c_{i_{2}}\left(\frac{\xi \left(x-x_{B,i_{2}}\right)}{x-x_{B,i_{2}}}\right)\right),$$
  (61) $$\frac{\partial \left({\tilde{\mathrm{PWL}}}_{1}\left(x,y\right)\right)}{\partial y}=1,$$
  (62) $$\frac{\partial \left({\tilde{\mathrm{PWL}}}_{2}\left(x,y\right)\right)}{\partial y}=1,$$

where $\left(x_{B,i_{j}},y_{B,i_{j}}\right)$ is the *i*-th breakpoint of the *j*-th $\tilde{\mathrm{PWL}}$ functions ${\tilde{\mathrm{PWL}}}_{1}$ and ${\tilde{\mathrm{PWL}}}_{2}$, respectively. Every expression presented in this section has been implemented in our *C*++ program. The symbolic manipulation reduces the computing time because it only requires substitutions and evaluations.

### 5.6. A Systematic Criterion to Select the Repulsion Parameter

The selection of repulsion parameters for obstacles has been an open problem since the HPPM was proposed in Reference [19]. This is a complex task because the effect of each parameter has a great impact on the homotopy surfaces and, hence, in the solution curve. Advances in the characterization and creation of a criterion have been reported in previous works, where: A study of the effect produced by the sign and magnitude of each parameter over the homotopy curve was introduced in Reference [10], nevertheless, the criterion employed to set them was only based on empirical knowledge. Then, Reference [20] presents a criterion based on random selection and segmentation of the map to enhance the performance of the planner and find the shortest path. Also, a grouping obstacle strategy to reduce the number of repulsion parameters in the formulation was presented in Reference [20]. This strategy is applied to reduce the complexity of parameter selection tasks for maps with a large number of obstacles. Next, a first approach of systematic criterion based on the distance between obstacles and the diagonal path to properly select the repulsion parameter was presented in Reference [37]. Showed the effectiveness of a systematic criterion using examples, nonetheless, its performance takes a higher portion of the total execution time. This section is devoted to explaining a new systematic criterion based on results reported in Reference [10,20,37] and the properties of auxiliary $\tilde{\mathrm{PWL}}$ functions proposed in this work. The main property of the formulation that has an effect over the repulsion parameter is denoted in the auxiliary function (33) which contains all mathematical representations of obstacles in the map. This function produces a scaling effect in the value of every repulsion parameter due to its absolute value terms. These reduce the effect of repulsion parameters in the vicinity of target-points and proportionally changing its value with the proximity between obstacles and any $\tilde{\mathrm{PWL}}$ function. From the manipulation of Equation (33)

(63) $$\begin{array}{c} f_{{\tilde{\mathrm{PWL}}}_{2}}\left(x,y\right)={\tilde{\mathrm{PWL}}}_{2}\left(x,y\right)\\ -\left(\sum_{i=1}^{i=n}\frac{p_{O,i}\left(\xi \left({\tilde{\mathrm{PWL}}}_{2}\left(x,y\right)\right)+\xi \left({\tilde{\mathrm{PWL}}}_{1}\left(x,y\right)\right)\right)}{O_{i}\left(x,y\right)+\left|O_{i}\left(x,y\right)\right|}\right), \end{array}$$

here, i=1,2,3,…n, *n* is the number of obstacles in the map; $O_{i}\left(x,y\right)$ is the mathematical representation of any *i*-th circular obstacle, and $p_{O,i}$ represents its respective repulsion parameter. Then, the effective value of the parameter is denoted as

(64) $$p_{\mathrm{eff},i}=p_{O,i}\left(\xi \left({\tilde{\mathrm{PWL}}}_{2}\left(x,y\right)\right)+\xi \left({\tilde{\mathrm{PWL}}}_{1}\left(x,y\right)\right)\right),$$

where $p_{\mathrm{eff},i}$ is the effective value of repulsion parameter in Equation (33) for each *i*-th obstacle. It can be noticed that the value of $p_{\mathrm{eff},i}$ dynamically varies according to SA tracking the solution path, then, only one value of $p_{\mathrm{base}}$ is selected for all circular obstacles. From the characterization presented in Reference [10], the next rank of magnitude for this parameter is employed in the case studies; $1\geq |p_{\mathrm{base}}|>0$ for circular obstacles and $100\geq |p_{\mathrm{base}}|>1$ for ellipsoidal obstacles. The expression (64) denotes that parameter magnitude of any obstacle decreases according to its proximity to any $\tilde{\mathrm{PWL}}$ function.

**Figure 14:**
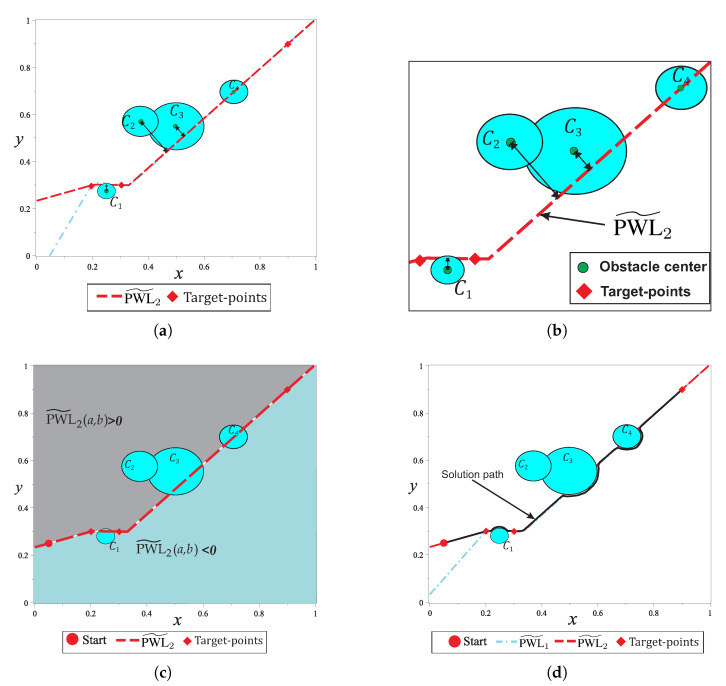
Criterion to select the repulsion parameter. (**a**) Environment map. (**b**) Position of obstacles with respect to ${\tilde{\mathrm{PWL}}}_{2}\left(x,y,\lambda \right)$. (**c**) Positive and negative regions with respect to ${\tilde{\mathrm{PWL}}}_{2}\left(x,y,\lambda \right)$. (**d**) Solution path using systematic criterion to select repulsion parameter.

In this subsection, an environment map with three circular obstacles is used to explain the proposed systematic criterion for selecting the sign of repulsion parameters. As already explained above, solution path tends to follow the function ${\tilde{\mathrm{PWL}}}_{2}\left(x,y\right)$ as a consequence of using a dummy obstacle. In this sense, the criterion to set the sign of the repulsion parameter for any circular obstacle can be established from its center position with respect to ${\tilde{\mathrm{PWL}}}_{2}\left(x,y\right)$ (see Figure 14b). This criterion is based on that, for all points (a,b) located above ${\tilde{\mathrm{PWL}}}_{2}\left(x,y\right)$, the value of ${\tilde{\mathrm{PWL}}}_{2}\left(a,b\right)$ is greater than zero; for points located below ${\tilde{\mathrm{PWL}}}_{2}\left(x,y\right)$, the value is less than zero (see Figure 14c). By using the dynamic value of repulsion parameter property provided by the formulation and relative position of the center $\left(\left(x_{C,i},y_{C,i}\right)\right)$ for every *i*-th circular obstacle with respect to ${\tilde{\mathrm{PWL}}}_{2}\left(x,y\right)$, a new definition of the repulsion parameter is described by

(65) $$p_{O,i}=\left\{\begin{array}{cc} -p_{\mathrm{base}}\mathrm{sgn}\left({\tilde{\mathrm{PWL}}}_{2}\left(x_{C,i},y_{C,i}\right)\right) & \mathrm{if}\;{\tilde{\mathrm{PWL}}}_{2}\left(x_{C,i},y_{C,i}\right)\neq 0,\\ -p_{\mathrm{base}} & \mathrm{if}\;{\tilde{\mathrm{PWL}}}_{2}\left(x_{C,i},y_{C,i}\right)=0, \end{array}\right.$$

where $p_{O,i}$ is the base parameter value with a sign that determines a lower or upper side path according to the *i*-th circular obstacle. Figure 14d shows the resulting solution path once the systematic criterion to set the repulsion parameter is executed. In this case study, the strategy is capable to properly set the signs of repulsion parameters which generate an efficient path with minimum length. This example validates the criterion effectiveness, which is addressed in the following sections.

The criterion introduced in this subsection has been formulated and characterized only for circular obstacles because of its symmetry. Nonetheless, it is assumed that ellipsoidal obstacles are only employed to represent walls or any other obstacle of big dimensions on the map. Then, the criterion to automatically assign the sign of repulsion parameter needs an additional step which will be explained in the next section.

## 6. Multiple-Target Homotopic Path Planning Method with Visibility Graph Approach

**Figure 15:**
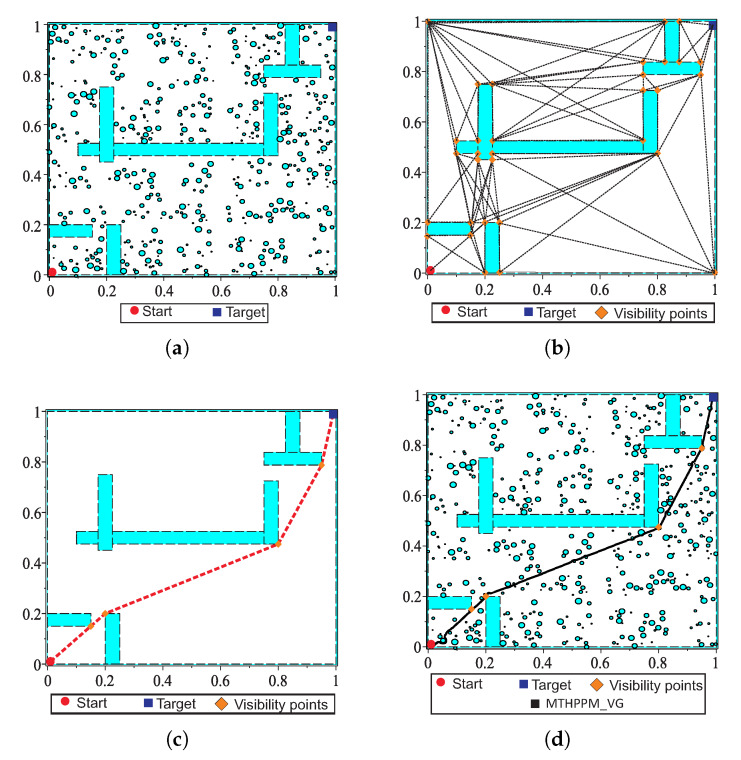
Visibility approach applied to Homotopic Path Planning Method. (**a**) Environment map with walls. (**b**) Visibility points and pre-processed path. (**c**) Visibility graph path without circular obstacles. (**d**) Solution path obtained by MTHPPM using the visibility approach.

Visibility graph (VG) is one of the most widely used roadmap methods to find the shortest path. This method employs a geometrical map representation to generate a roadmap that contains all links between vertices of the polygonal obstacles, start-point, and target-point [12,13,14]. Although this algorithm can find the shortest path for maps of structured environments, it is only employed to solve configurations with few polygonal obstacles, because its execution time and memory consumption depends on the number of obstacles [12]. In this work, the visibility graph algorithm is applied to pre-process the map and obtain a first approximation of the solution path for closed and office-like environments. In this way, only big polygonal objects such as walls and office furniture (static objects) are considered. Then, MTHPPM uses the path provided by VG (commonly the shortest path) to compute a collision-free path for the full environment containing non-polygonal obstacles. Figure 15 shows the operation process of MTHPPM using the visibility approach applying (MTHPPM_VG) for an office-like environment with five hundred obstacles.

For the example in Figure 15a, the main task of the mobile robot is to reach the target-point located on the opposite corner of the room, concerning to the initial position. Solving this example is done through the following steps; first, an approximation of the solution path is obtained by the visibility graph algorithm using a map, where only walls are considered. The configuration map without obstacles is reduced to a simple problem (see Figure 15b). The graph complexity is further reduced using the premise that $\tilde{\mathrm{PWL}}$ formulation is a single-valued function. In this regard, every path with links that implies returns in *x*-axis is suppressed. Second, the first approximation of the path will be integrated by only links (corners of static obstacles and walls) in *x*-axis forward direction, as it is shown in Figure 15c. It can be noticed that, for environment maps with bug traps and maze layout, a path with visible vertices in forward direction might not exist. Two case studies with this problem are treated and solved in the next section. Third, the solution path provided by VG is employed to generate $\tilde{\mathrm{PWL}}$ formulation which contains the information about the position of the ellipsoidal obstacles. Then, automatic sign assignation for this type of obstacle is executed, operating in the same way as circular obstacles. The function to obtain the sign of an ellipsoidal obstacle is

(66) $$p_{O,i}=\left\{\begin{array}{cc} -p_{\mathrm{base}}\mathrm{sgn}\left({\tilde{\mathrm{PWL}}}_{2}\left(x_{R,i},y_{R,i}\right)\right) & \mathrm{if}\;{\tilde{\mathrm{PWL}}}_{2}\left(x_{R,i},y_{R,i}\right)\neq 0,\\ -p_{\mathrm{base}} & \mathrm{if}\;{\tilde{\mathrm{PWL}}}_{2}\left(x_{R,i},y_{R,i}\right)=0, \end{array}\right.$$

where $p_{O,i}$ is the base parameter value with a sign that determine if the solution path pass below or above the *i*-th ellipsoidal obstacle with center at point $\left(x_{R,i},y_{R,i}\right)$ and $p_{\mathrm{base}}$ is the base value selected, arbitrarily, for all ellipsoidal obstacles; from empirical data presented in Reference [10], the value is in range $100\geq |p_{\mathrm{base}}|>1$. Finally, the MTHPPM is executed for the configuration map containing obstacles, walls, and vertices of the visibility path as target-points; solution is the shortest collision-free path (see Figure 15d). The approach explained in this section can be used for problems with only one target-point or multiple-targets. For multiple-targets cases, the visibility points should be integrated in the set of targets as long as these do not represent a backward advance in the $\tilde{\mathrm{PWL}}$ formulation, in which case these points must be removed.

**Figure 16:**
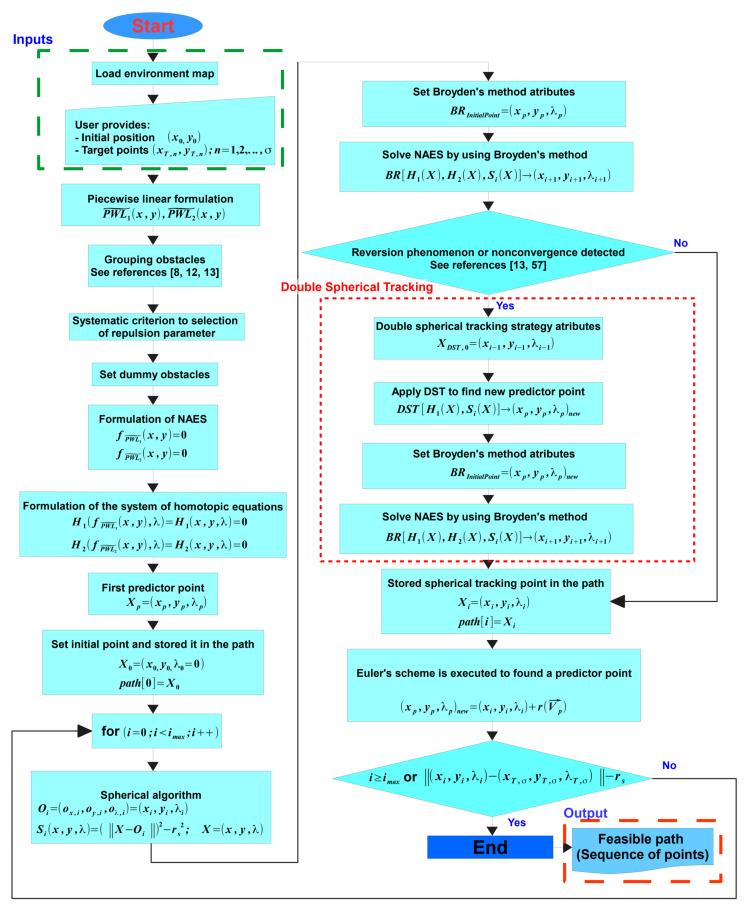
Multiple-target homotopic path planning method flow chart.

The procedure of the proposed multiple-target planner can be summarised using the flow chart presented in Figure 16. If the planning problem has only a single target the method is named HPPM and for multiple targets MGHPPM. Furthermore, when the planning problem is performed on a structured workspace with a single target point, the procedure is the same and the visibility graph approach is used to calculate the first approximation of the solution, and the homotopy based method is named MGHPPM_VG.

The flow chart (see Figure 16) contains all strategies explained previously and the addition of points generated by the visibility graph approach into the set of target-points. The proposed methodology considers that the environment map is known in advance or it is provided by sensors onboard (for real-time implementations) in the load environment map stage. In this sense, inputs in the proposed planner are a semi-algebraic representation of the environment map ($C_{\mathrm{space}}$), the initial position of the robot, and one or more target-points designed by the user or generated by a visibility-graph algorithm (because visibility graph planners have been treated strongly in others works such as References [12,13,14], in this work it is not integrated in the homotopic planner process, is only used as sub-process). Here, it is considered that the robot has a navigation module and control rules to guarantee the correct path following. Then, the iterative procedure of MTHPPM (for multiple-target points) or HPPM (for single target-point) starts. First, the mathematical model of the environment and data provided by the user is generated in the $\tilde{\mathrm{PWL}}$ formulation stage. Second, the techniques to enhance convergence, length of path, and automatic assignation of repulsion parameters are performed (grouping obstacles, systematic criterion to select repulsion parameter, and dummy obstacle addition). Third, homotopy system of non-linear equations to represent the configuration space is formulated in NAES formulation stage. Then, the first predictor point for the HPPM (or MGHPPM) is calculated from the initial position of the robot (first point of the path). Fourth, the iterative procedure of spherical algorithm is executed; within it, the predictor-corrector scheme and reversion phenomenon (or non-convergence) check are performed for each step. This procedure is executed as explained in Section 3, where the Broyden’s method is employed to calculate a new point of path which represents a solution of the system of homotopic equations and Euler’s scheme is executed to obtain a new predictor point. During the Spherical algorithm procedure, if a reversion or non-convergence is detected, the DST technique is enabled and employed to solve this issue as already explained in the Section 5.3. Finally, the procedure finishes when the SA reaches a target-point or the maximum number of steps is achieved; then, returns a set of points that represent the solution path. In the next section, some case studies are presented to validate the utility of this methodology.

## 7. Case Studies

This section provides five case studies to provide certainty about the usefulness of the proposed methodology and how it solves path planning problems like bug traps and narrow corridors. The first three simulations are focused on how to solve planning problems in sceneries with narrow corridors. Simulation four demonstrates its effectiveness to solve maps with bug traps. On the one hand, for these four case studies, a comparison between the proposed methodology in its two variants (HPPM formulation and MTHPPM with visibility graph approach) and six sampling-based planning algorithms is provided. This comparison is based on important aspects like: memory consumption, execution time, percentage of feasible paths found, and path length. On the other hand, an example of multiple-target path planning for a closed environment with narrow corridors is shown in simulation five. All simulations were performed using the following set-up: all planners have the same step size (spherical tracking radius for MTHPPM and HPPM; collision checking resolution parameter for BSP algorithms), MTHPPM code in C++, and executed on PC (Intel i7 2.6 GHz processor, RAM 16 GB, and 64-bit Ubuntu 16.04 operating system). SBP algorithms ran on the same PC using the well known Open Motion Planning Library v.1.3.1 [57] and OMPL Planer Arena [58] to obtain performance data. To highlight advantages and weaknesses of the proposed homotopy based planner, it is compared to eight SBP algorithms: Expansive Space Trees (EST), Kinematic Planning by Interior-Exterior Cell Exploration (KPIECE1), Probabilistic Roadmap Method (PRM), Rapidly-exploring Random Trees (RRT), Bidirectional Rapidly-exploring Random Trees (Bi-RRT, also named RRT-Connect), Rapidly-exploring Random Trees with A* approach (RRT*), RRT and PRM with Lazy collision method (LazyRRT), and (Lazy PRM). To obtain a significant performance data, each SBP algorithm was run one hundred times.

### 7.1. Case 1

**Figure 17:**
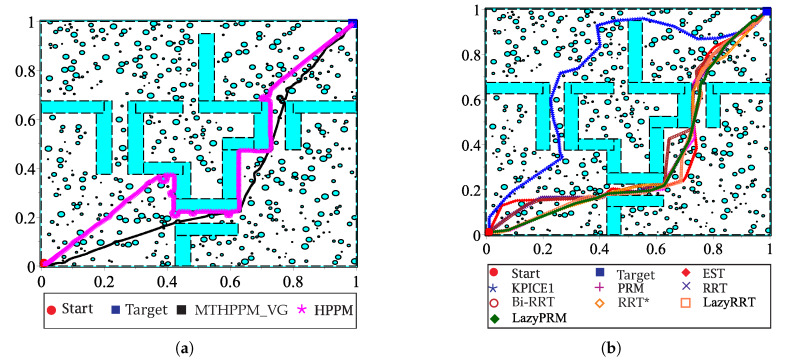
Visual comparative between MTHPPM and HPPM against best run of each SBP algorithm for case study 1. (**a**) Paths obtained with HPPM and MTHPPM_VG. (**b**) Paths obtained with SBP algorithms.

This case study is devoted to show the ability of MTHPPM to deal with the narrow corridor problem. Figure 17a depicts the visual comparison between HPPM and MTHPPM_VG (MTHPPM with visibility graph approach) paths on a normalized office-like map in 2-D with five hundred circular obstacles. Here, the initial position of the robot is at point (0,0) and target-point at (1,1). The visual comparison between paths obtained by SBP algorithms is presented in Figure 17b.

**Figure 18:**
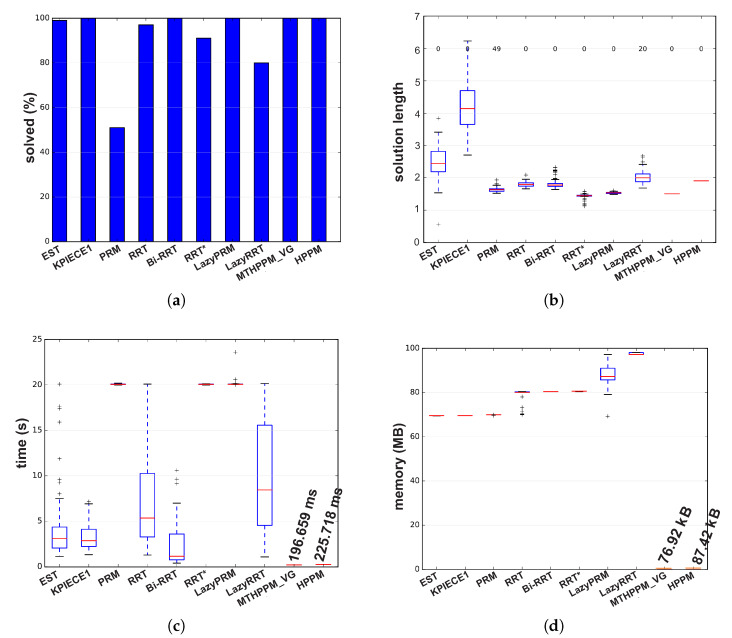
Comparative results for case study 1. (**a**) Successful paths. (**b**) Path length. (**c**) Execution time. (**d**) Memory.

Figure 17a,b, the calculated paths by HPPM against MTHPPM_VG and SBP algorithms (shortest path after 100 runs) are contrasted. It is important to note that the path obtained using HPPM is one of the longest because it tends is to round all obstacles in direction to the diagonal line between initial and target-point. On the other hand, the path obtained by MTHPPM with visibility approach is one of the shortest. Figure 17a shows the effectiveness of HPPM and MTHPPM (MTHPPM_VG) to solve maps with narrow corridors and several obstacles. Furthermore, the ability of MTHPPM to calculate the shortest collision-free path using the visibility graph approach explained in Section 5 is validated in this case study. The box plots (box-and-whisker diagrams) in Figure 18 depicts a quantitative comparison between HPPM, MTHPPM_VG, and SBP algorithms for percentage of feasible paths found, execution time, path length, and memory consumption.

Figure 18 shows the summarized results which denote the following characteristics: First, Figure 18a shows that percentage of fails for PRM is higher than twenty percent, while for LazyRRT is closer to that percentage. For HPPM and MTHPPM_VG, the success rate is one hundred percent due to its deterministic formulation. Second, the execution time spent to solve the configuration map is presented in Figure 18c. In this box-and-whisker diagram, it can notice, that SBP algorithms are in the order of seconds, while MTHPPM_VG and HPPM their time is in the order of milliseconds. Third, the results in Figure 18b show that the path obtained by MTHPPM_VG is one of the shortest, RRT* provided the best. Finally, memory consumption comparison exhibits a big gap between HPPM and MTHPPM against SBP algorithms. The difference is about three orders of magnitude, from KB to MB. This is because SBP algorithms store a roadmap of collision-free points, while homotopy based methods only store the path and obstacle positions.

### 7.2. Case 2

**Figure 19:**
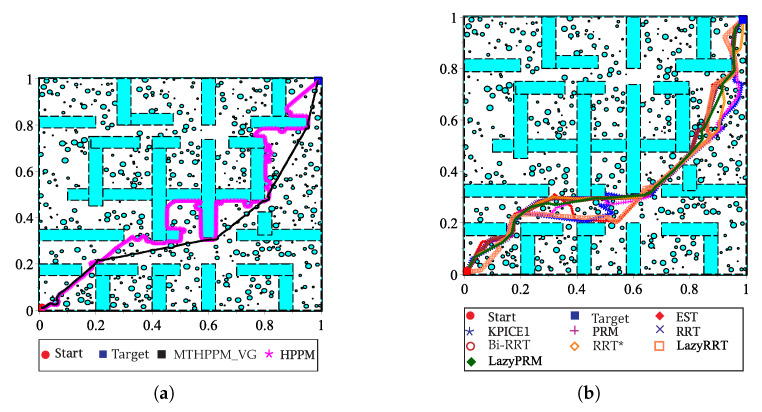
Visual comparative for case study 2 with five hundred obstacles; best run of each SBP algorithm. (**a**) Paths obtained with HPPM and MTHPPM_VG. (**b**) Paths obtained using SBP algorithms.

This case study presents a path planning problem of an office-like environment with narrow corridors and five hundred obstacles. This example shows one of the future applications of HPPM and MTHPPM, to integrate it in the navigation system of a parcel service robot. The main task of the robot, in this example, is to collect and deliver packages or documents from one cubicle to another. Figure 19a provides the floor plan of an office represented on a normalized 2-D space, where, the initial and target points are located in opposites corners.

**Figure 20:**
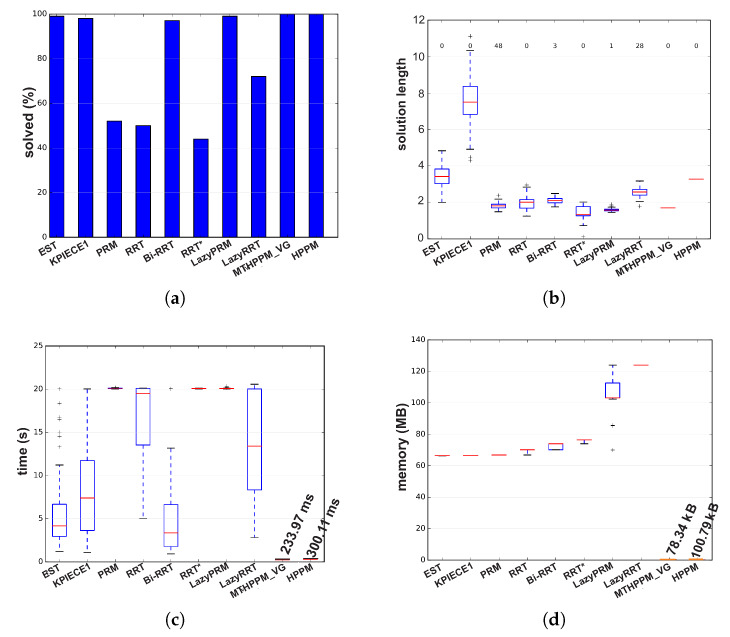
Comparative results for case study 2. (**a**) Successful paths. (**b**) Path length. (**c**) Execution time. (**d**) Memory.

Figure 19a,b show a visual comparison of paths generated by HPPM against MTHPPM_VG and between SBP algorithms, respectively. In these patterns, the difference between path lengths calculated by SBP algorithms, HPPM, and MTHPPM_VG are depicted. It is important to note that, for SBP algorithms, the most optimistic simulation is taken, that is, the shortest path after one hundred runs. Figure 20 shows the box-and-whisker diagrams of the performance results for SBP algorithms and homotopy based planners (HPPM and MTHPPM).

From simulations, it can be concluded the following: First, RRT, PRM, RRT*, and LazyRRT have a poor (less than eighty percent) performance of success percentage metric against the homotopy based planners. Second, like the results of the previous case study, HPPMs spent less time, significantly, than sampling-based planners (see Figure 20c). Third, meanwhile HPPM found one of the longest paths, MTHPPM_VG obtained one of the three shortest; and the best compared to LazyPRM and RRT*. Finally, memory used by homotopy based planners is about a thousand times smaller than the sampling algorithms, this is due to the formulation of each approach.

### 7.3. Case 3

**Figure 21:**
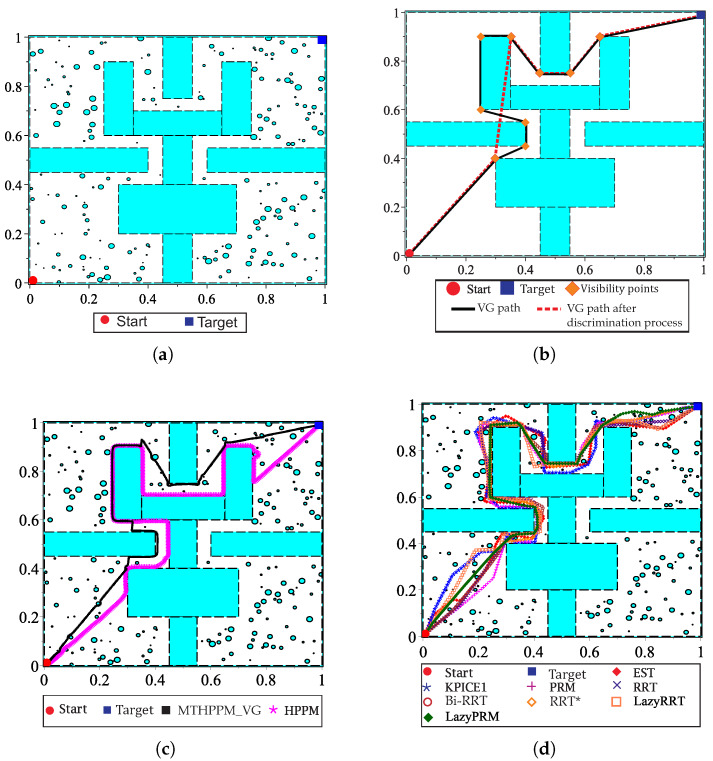
Comparative results for case study 3; best run of each SBP algorithm. (**a**) Path planning problem case study 3. (**b**) Visibility graph path without circular obstacles. (**c**) Paths obtained with HPPM and MTHPPM_VG. (**d**) Paths obtained with SBP algorithms.

This case study presents an example in which some visibility points are selected to create a forward direction sequence of target-points. This is adequate to be integrated in a single-value piecewise linear formulation employed in MTHPPM, as already explained in Section 5. Figure 21a depicts the environment map for this case, contains walls, and two hundred circular obstacles. The operation of the proposed MTHPPM_VG methodology for this configuration is similar to previous examples, furthermore, the visibility-points selection process is added. First, the approximation of the solution path is computed using the VG algorithm, then, an automatic criterion to discard nodes of the visibility path which implies backward advance in *x*-axis. Figure 21b shows nodes and paths after the visibility-point selection process is executed; the remaining nodes are employed as target-points for MTHPPM (dashed path). Figure 21c,d displays the resulting collision-free paths for HPPM against MTHPPM and between SBP algorithms (shortest path after one hundred runs for each SBP), respectively.

**Figure 22:**
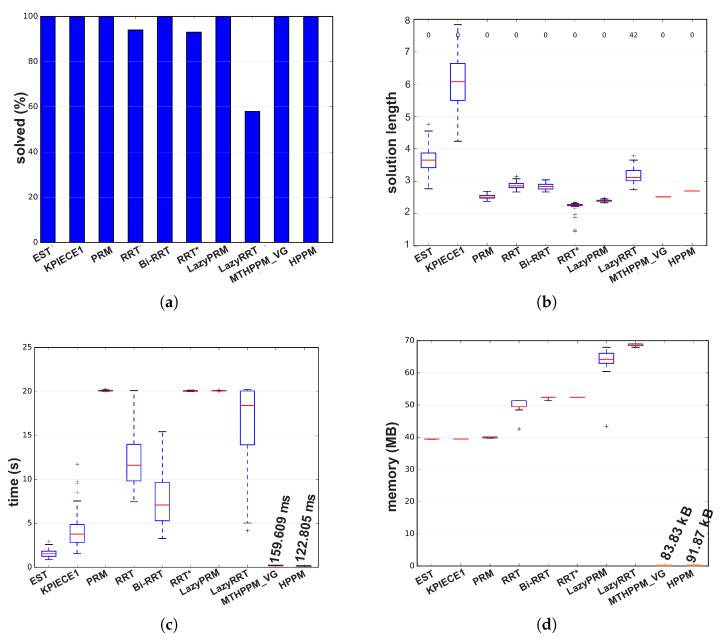
Comparative results for case study 3. (**a**) Successful paths. (**b**) Path length. (**c**) Execution time. (**d**) Memory.

Figure 21 shows that the path obtained by MTHPPM_VG is drastically shorter than the HPPM path. Figure 22 show the summarized results of HPPM, MTHPPM, and SBP algorithms for the most significant metrics.

By analyzing the simulations, it is important to note: First, lazyRRT has the worst performance of all SBP algorithms concerning to percentage of feasible paths found; EST, KPIECE1, PRM, Bi-RRT, LazyPRM have one hundred percent of success. Second, using results from previous case studies, homotopy based planners spend, notoriously, the shortest time compared to sampling-based planners (see Figure 22c). Third, MTHPPM_VG obtains a path very close to the shortest (RRT* calculated the best). Finally, consumed memory by homotopy based planners are around one thousand times lower than SBP algorithms.

### 7.4. Case 4

**Figure 23:**
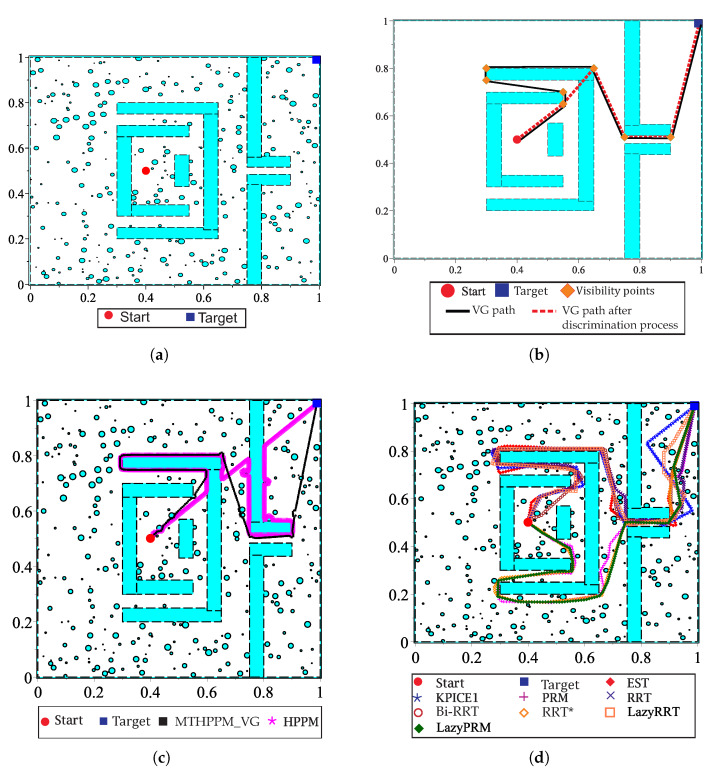
Comparative results for case study 4; best run of each SBP algorithm. (**a**) Path planning problem case study 4. (**b**) Visibility graph path without circular obstacles. (**c**) Paths obtained with HPPM and MTHPPM_VG. (**d**) Paths obtained with SBP algorithms.

This case study focuses on the bug trap problem, additionally, it deals with visibility nodes issue introduced in the previous case study. Figure 23a shows the configuration map with three hundred circular obstacles, walls, and narrow corridors. The initial position of the robot is inside of the bug trap, then, the planners need to plan a path that allows them to surround it, avoid the trap, and pass through two narrow corridors to reach target-point placed at the corner on the map. Similar to the previous example, for MGGHPPM, the VG algorithm found a first approximation of the path considering only the walls. Afterward, the discrimination process is executed to delete nodes of the VG path, which implies tracking in the backward direction as drawn in Figure 23b (dashed path). Finally, the full map is solved by MTHPPM using the remaining nodes of VG path as target-points. The resulting collision-free paths for HPPM against MTHPPM and between SBP algorithms (shortest path after one hundred runs for each SBP) are presented in Figure 23c,d, respectively.

**Figure 24:**
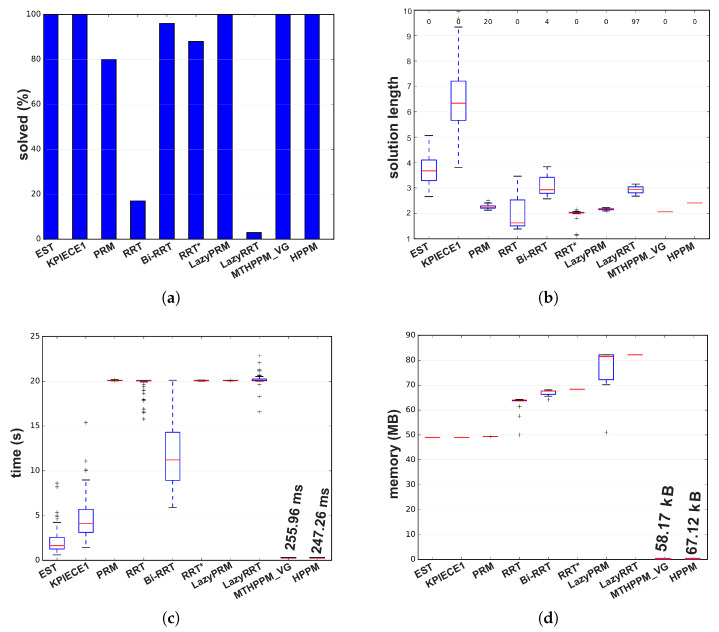
Comparative results for case study 4. (**a**) Successful paths. (**b**) Path length. (**c**) Execution time. (**d**) Memory.

Figure 23c shows the ability of HPPM and MGHPPM_VG to deal with a bug trap and narrow corridors. The tendency of HPPM to follow a direct path can be observed in Figure 23c. On the other hand, it also depicts an enhanced homotopy path since MTHPPM uses the points provided by the VG algorithm. Figure 24 shows a condensed performance information of the HPPM, MTHPPM, and SBP algorithms for time consumption, memory, percentage of feasible paths found, and length of the path.

Analyzing the obtained results, can be summarized as follows: First, RRT and LazyRRT exhibit poor performance to deal with bug traps; unlike EST, KPIECE1, PRM, HPPM, and MTHPPM_VG which have a one hundred percentage of success (see Figure 24a). Second, Figure 24c shows that homotopy based planners spent, noticeably, less execution time than sampling-based planners. Third, the HPPM creates a path with competitive length, while MTHPPM_VG obtained a path close to the shortest (RRT* calculated the best). For this metric, the results of RRT because the length of its best path corresponded to an unsuccessful run are discarded. Finally, homotopy planners exhibited execution times around three orders of magnitude lower than the best SBP algorithms.

### 7.5. Case 5

**Figure 25:**
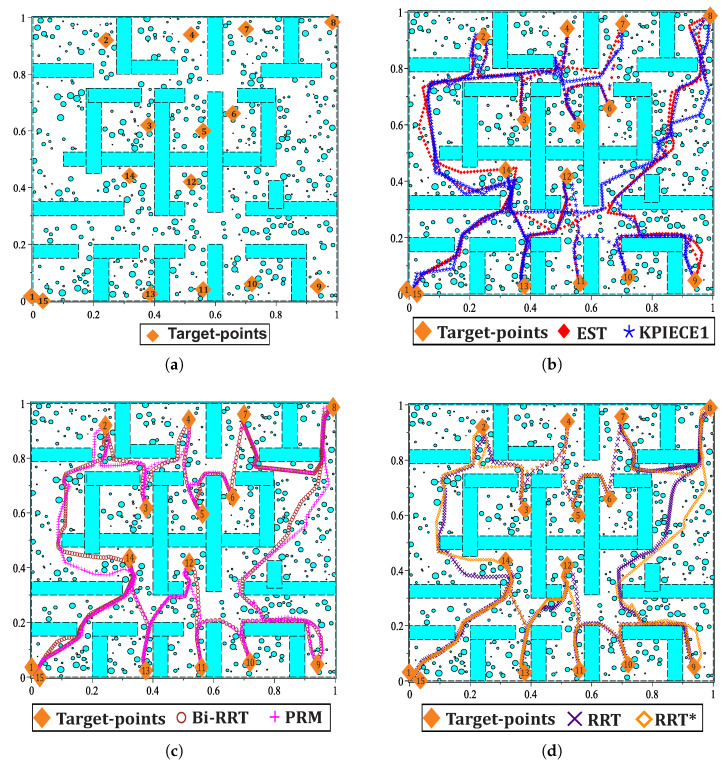
Multiple-target path problem solution using SBP algorithms with point-to-point strategy. (**a**) Multiple-target point problem (case study 5). (**b**) Multiple-target path using EST and KPIECE1. (**c**) Multiple-target path using RRTConnect and PRM. (**d**) Multiple-target path using RRT and RRT*.

The utility of MTHPPM to solve the problem of multiple-target path planning in applications like pick-and-delivery activities, museum guidance robot, rescue task robot, and so forth, is presented. For this case study, target-points are previously selected by the user according to a sequence of rooms that the robot must visit. Figure 25a shows the environment with a sequence of fifteen milestones, including the start-position (point 1) and target-position (point 15). This map contains five hundred circular obstacles representing the configuration of people (considered as static obstacles for a given instant of time). The main objective of this case study is to show the ability of MTHPPM to calculate a collision-free path for multiple-targets in a map filled with obstacles. For this case, each SBP algorithm was executed one hundred times and applied the point-to-point strategy to generate a path to visit all fifteen target-points. Figure 25 depicts the solution paths of six SBP algorithms generated using the point-to-point technique. These figures show the best run (length of path) for each SBP algorithm between two points after one hundred runs.

**Figure 26:**
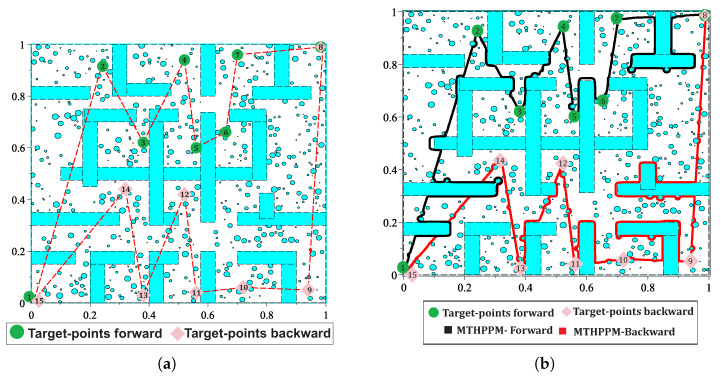
Performance of MTHPPM for office-like environments. (**a**) Piecewise-linear representation of direct path for multiple-target approach. (**b**) Successful solution path.

Figure 25b shows that EST and KPIECE1 planners are not capable to solve the problem of the narrow corridor between target-points seven and eight. Here, it presents a visual comparison of full path length because benchmark results provided a similar outcome regarding memory consumption, time, and success percentage compared to previous experiments. It is important to remark that, the full path of each SBP algorithm is integrated by the shortest segments between two points obtained after one hundred executions. Figure 26a presents the single value $\tilde{\mathrm{PWL}}$ function employed to calculate two multiple-target paths through MTHPPM. The fist path is calculated for the motion in the forward direction (path from point 1 to 8) and the second in the backward direction (path from point 8 to 15), as can be seen in Figure 26b.

Figure 26b shows the ability of MTHPPM to obtain a free collision path for a sequence of target-points. This planner calculates the full path in only two runs, one path in the forward and the other one in the backward direction. Although the path obtained by MTHPPM is not optimal, this case study shows the application of the proposed MGHPPM to solve multiple-target problems with hundreds of obstacles and narrow corridors. This characteristic is desirable for monitoring robots in oil platforms and forests or natural reserves.

## 8. Pure-Pursuit Controller Using Matlab Robotics System Toolbox

**Figure 27:**
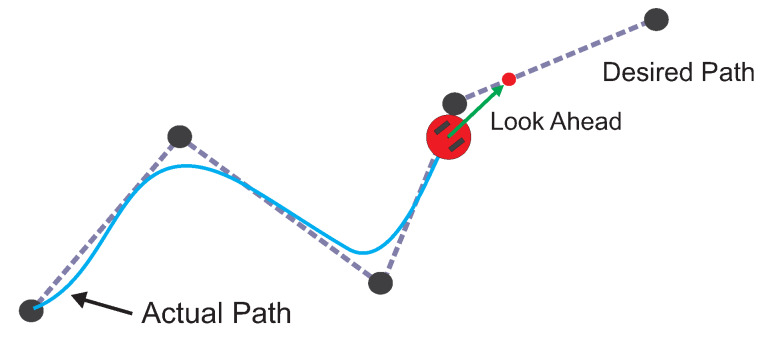
Pure-pursuit scheme.

This section provides two simulations to validate the feasibility of paths obtained by HPPM, MTHPPM, and the pure-pursuit algorithm. The pure-pursuit algorithm procedure computes the angular velocity of the robot from the current position to reach a look-ahead point (see in Figure 27). This is not a common controller, although it is suitable to be employed for path tracking purposes [59,60]. Here, two tracking simulations of a waypoints set obtained by homotopy planners (HPPM and MTHPPM) are used. The tracking is simulated by the pure-pursuit class contained in MATLAB Robotics System Toolbox and the model of a differential drive robot. It is assumed that linear velocity is constant and angular velocity changes according to the instantaneous center of curvature (ICC). The ICC is calculated from the look-ahead parameter as explained in References [59,60]. Pure-pursuit operates as follows: First, the desired path is obtained by any path planning algorithm, a homotopy planner for this case. The path should be represented as a finite array of *n*-waypoints in the form ($x_{i},y_{i}$), i=1,2,3,…,n. Second, using the look-ahead distance (value previously set), the algorithm calculates ICC and then the angular velocity to move the robot from one waypoint to another until the last point of the path is reached.

Figure 27 presents the basic operation of the pure-pursuit controller. It can be noticed that the look-ahead distance shown in this figure is shorter than the distance between two waypoints. For these cases, the algorithm completes the desired path using segments of straight lines. For the next simulations, parameters of pure-pursuit controller are set as follows

- The dimensions of the environment map are 15 m × 15 m.
- Robot radius is 0.25 m.
- Look-ahead distance is 0.5 m.
- Linear velocity is 0.5 m/s.
- Maximum angular velocity is 2rad/s.

**Figure 28:**
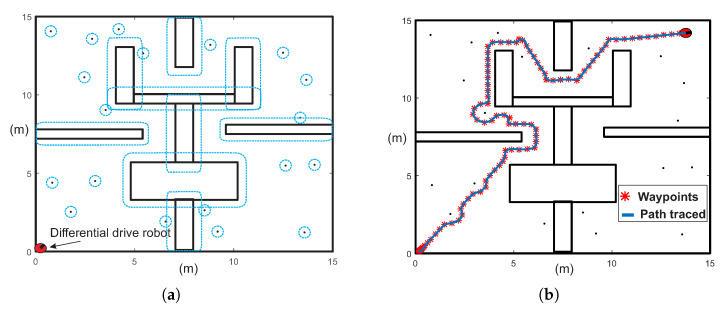
Pure-pursuit path tracking for the solution path of case study 3. (**a**) Floor plan considering dimensions of robot. (**b**) Path traced by differential drive robot.

**Figure 29:**
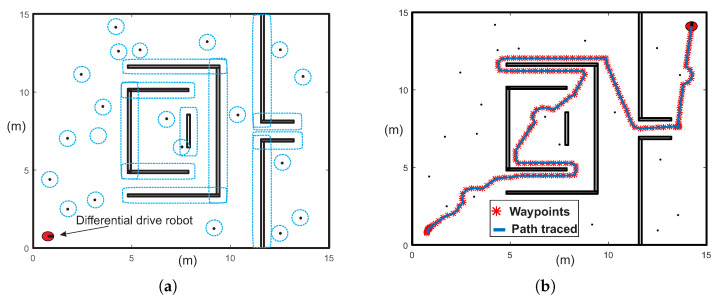
Pure-pursuit path tracking for the solution path of case study 4. (**a**) Floor plan considering dimensions of the robot. (**b**) Path traced by differential drive robot.

In this example a configuration similar to case studies 3 and 4 in Section 7 is applied. Before the homotopy based planner is executed, the dimensions of the robot must be added to all obstacles, the robot dimensions are considered, as shown in Figure 28a and Figure 29a. In these, the differential drive robot is modeled by a disc, then, its radius is added to dimensions of all obstacles as guard distance (dash line in Figure 28a and Figure 29a), as explained in Reference [10]. The complete environment map, considering dimensions of the robot, is normalized to be introduced in HPPM. Figure 28b and Figure 29b show the path followed by differential robot employing the pure-pursuit algorithm. These figures show that the difference between waypoints (homotopy path) and pure-pursuit path is minimal.

For the example of Figure 28, the initial point is placed at (0,0) and final point at (14.5m,14.5m). Meanwhile, for the example of Figure 29, the initial point is placed at (0.5m,0.5m) and final point at (14.5m,14.5m).The apparent radius (dash line) of all circular obstacles is 0.26 m, which represents the sum of the obstacle radius (0.01 m) and the robot radius. The capability of HPPM to find solution of maps with narrow corridors can be observed in the example of Figure 29b.

Figure 28 and Figure 29 validate that paths obtained by the proposed homotopy based planner (MGHPPM) can be applied to differential drive robots through the pure-pursuit controller. These simulations also denote that homotopy paths do not require post-processing or smoothed unlike SBP algorithms.

## 9. Discussion

This work presents a novel planner to obtain a collision-free path from a sequence of target-points which contains several improvements in the numerical implementation of the original homotopic path planner [19]. The main contributions of this work are outlined in four aspects.

1. A novel NAES formulation based on smooth PWL auxiliary functions ($\tilde{\mathrm{PWL}}$) generated from and approximation of absolute values function is presented. This formulation allows generating a multiple-target planner scheme, the MTHPPM, which uses the ability of the continuation homotopy to find more than one solution. Furthermore, the $\tilde{\mathrm{PWL}}$’s provides a scheme without mathematical discontinuities due to the integration of approximation of absolute value on the PWL formulation. For this scheme, the targets can be set by the user or using an automatic process, as explained in the case studies. Furthermore, this formulation does not imply a significant increase in execution time or memory consumption respect to original HPPM.
2. A dummy obstacle scheme to reduce the number of steps needed to generate a successful path was proposed. This scheme generates a modification in one of the homotopy surfaces, which reduces the distance of the solution path (points of intersection between homotopy surfaces). The effect of a dummy obstacle has a great impact on the number of steps in the procedure of homotopy based planner (HPPM and MTHPPM), thus, the execution time is significantly reduced.
3. The operation of the technique to solve the reversal phenomenon, found in spherical tracking, named double spherical tracking (DST) is presented and explained in Section 5.3. This technique also can be applied to improve the convergence of SA when the corrector scheme (Broyden’s method) fails. The effectiveness of this technique is validated by numerical simulations presented in Section 7, in which, DST technique allowed the continuation of path planning. Table 2 presents the issues (reversal phenomena and non-convergences of corrector scheme), steps in which were detected, and a total of steps (number of points in the path) for case studies 1–4. These data validate the effectiveness of DST technique to solve the reversal phenomenon and enhance the convergence of HPPM and MTHPPM (MTHPPM_VG). The non-convergence and reversal effect issues during the iterative HPPM and MTHPPM processes for case studies 1–4 were between 0.082–0.633% of the total steps, that is, the impact over the execution time and memory consumption is hardly noticeable. It is important to remark that, although MTHPPM (MTHPPM_VG) is more susceptible to non-convergence and reversal effect issues than HPPM because of its formulation, the implementation of multiple-target strategy reduces the number of SA steps and enhance the homotopy path (in terms of length).
4. Automatic assignation of sign and magnitude of repulsion parameter for circular obstacles is introduced in Section 5.6. This formulation optimizes paths length because it forces the homotopy curves to stay close at the direct trajectory.
5. The multiple-target HPPM with visibility graph approach is provided in Section 6. For this method, a first approximation of the path is obtained by visibility graphs algorithm considering only walls in the environment, then, the visibility points (nodes of visibility graph path) are taken as targets by MTHPPM and solve the entire map for all obstacles and walls. This approach offers the best of both methods (Visibility graph and MTHPPM): (a) Shortest path (from visibility graph), (b) low time and memory consumption (from MTHPPM), and (c) ability to find the solution path if it exists (from the combination of MTHPPM and VG).
6. The symbolic manipulation of the Jacobian matrix proposed in this work is employed to simplify the complexity of the homotopy based planner implementations. This strategy allows a fast evaluation of the Jacobian matrix in the predictor and corrector schemes (process implicit at each SA step). Furthermore, this strategy avoids the use of specialized mathematical libraries and packages and provides an easy and cheap (in terms of memory) implementation in any programmable platform.
7. The feasibly of paths obtained with the MTHPPM and HPPM to be executed by a differential drive robot model is shown in Section 8. The simulations presented here proved that homotopy paths have a great compatibility with the pure-pursuit scheme due to the smoothness of the paths. Furthermore, Figure 28b and Figure 29b showed, visually, that difference between homotopy path (waypoints) and traced path (using pure-pursuit controller) is very small. It implies that the homotopy planner does not need an additional stage of post-processing due to the smoothness of the paths, unlike the SBP algorithms which need an additional process to simplify the path found [2,3,7,8,9,10].

Table 3 shows the summarized results of case studies 1–4 for the maximum, minimum, and median values of performance metrics presented in its respective box diagrams. It is important to note that minimum length (dimensionless because map is normalized) for some SBP planners denotes the result of an unsuccessful run. Furthermore, this table shows the global execution time for all SBP algorithms, which considers the execution time of the algorithm and time spent to smooth the path (path simplification process). If the median value of each metric for every SBP planner is taken, then, it is possible to remark the following conclusions for the case studies: (I) The HPPM and MTHPPM have the best performance for memory consumption, about one-thousandth, compared to the amount spent by SBP algorithms; for instance, lazzyRRT and LazzyPRM for the second case study. (II) The execution of homotopy based planners is between ten and one hundred times faster than SBP algorithms. (III) The homotopy based planners presented in this paper have an adequate balance between the measured performance metrics; while for SBP algorithms, execution time and length of the path are inversely proportional parameters. In other words, faster planners (EST, KPIECE1, and RRT) find longer paths and shortest path calculations consume the maximum amount of time (RRT*). Results also show that the proposed methodology does not have any dependence on libraries, packages, or external functions because complex procedures are treated using symbolical analysis as it is presented in Section 5.5. Therefore, its implementation is simple and cheap in terms of computational resources. This allows an easy implementation in multiple platforms like embedded systems with microcontrollers, microprocessors up to implementations in PC’s and FPGA’s. In addition to these advantages, simulations presented in Section 8 show that the path obtained through homotopy based planners can be easily followed by a differential drive robot using the pure-pursuit algorithm.

**Figure 30:**
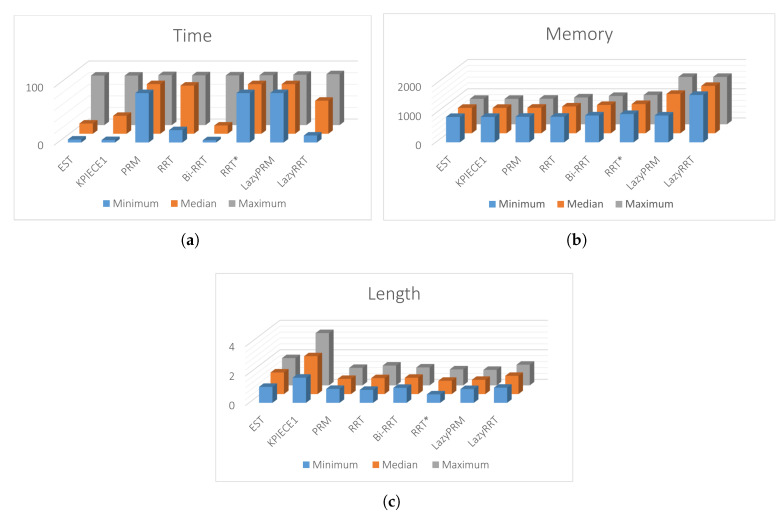
Bar graphs for time, length of path, and memory in number of times respect to the MGHPPM results of case 1. (**a**) Execution time. (**b**) Memory. (**c**) Length of path.

**Figure 31:**
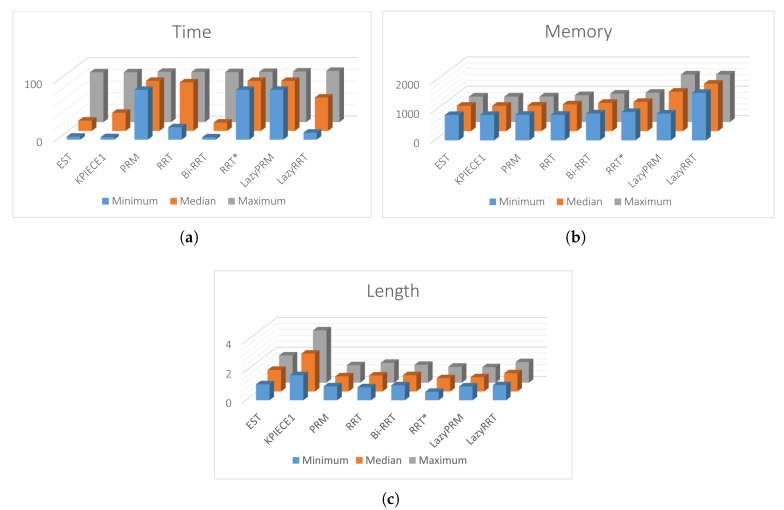
Bar graphs for time, length of path, and memory in number of times respect to the MGHPPM results of case 2. (**a**) Execution time. (**b**) Memory. (**c**) Length of path.

**Figure 32:**
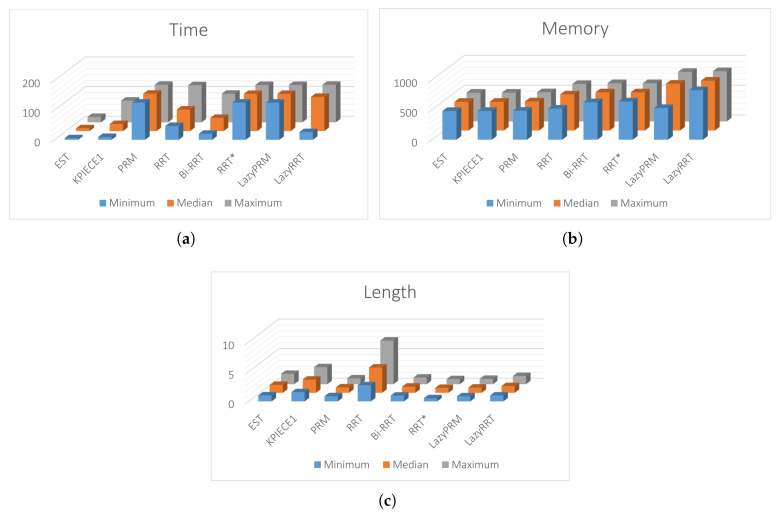
Bar graphs for time, length of path, and memory in number of times respect to the MGHPPM results of case 3. (**a**) Execution time. (**b**) Memory. (**c**) Length of path.

**Figure 33:**
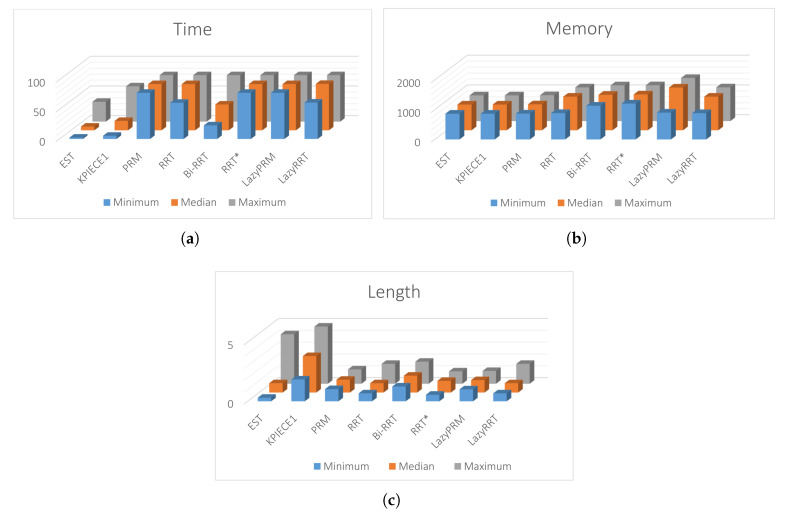
Bar graphs for time, length of path, and memory in number of times respect to the MGHPPM results of case 4. (**a**) Execution time. (**b**) Memory. (**c**) Length of path.

Figure 30, Figure 31, Figure 32 and Figure 33 show the minimum, median and maximum results for execution time, length of path, and memory of cases 1–4 (in number of times), according to the results of MGHPPM (MTHPPM_VG). These figures represent a visual interpretation of Table 3 which helps to denote the advantages of the MGHPPM where, a) the memory used in all case studies by the SBP algorithms is between hundreds and thousands of times greater than MGHPPM; b) the path obtained through MGHPPM is very close to the shortest path found by SBP algorithms, and c) the execution time of MGHPPM for all case studies is between five and one hundred of times smaller than every SBP algorithms.

## 10. Conclusions and Future Work

In this work, a path planning method with multiple-target applications has been presented, it is capable of solving complex maps of hundreds of obstacles. This method contemplates in its computational core a series of novel and effective tools such as Double Spherical Tracking, the integration of a dummy obstacle to improve performance and reduce computing time and the number of iterations. As well as, a scheme of multiple solutions of a NAES formulated by approximations of PWL functions. In the same way, two new schemes are proposed to avoid discontinuities in the spherical tracking using patches and bridges in the plane. Furthermore, a simple and efficient solution is proposed as a criterion for the automatic selection of the repulsion parameter of the obstacles representation for the homotopy based planner methods.

From the case studies it can be denoted first, the proposed planner (MTHPPM) is faster; between five and one hundred times than the average of one hundred runs for each SBP algorithms. Second, the calculated path is very close to the shortest path; the difference is between ten and twenty percent. Third, the success rate is one hundred percent for all case studies, while some SBP algorithms achieved around thirty-five percent of the failure rate. Fourth, the proposed planner uses just one-thousandth of the memory than the best SBP algorithms employs for every case study. Fifth, the proposed methodology does not have any dependence on libraries, packages, or external functions for which its implementation is simple as well as cheap in terms of memory and computational resources. Therefore, these characteristics allow its implementation for real-time applications in multiple platforms from embedded systems with microcontrollers of low resources, such as it is presented in Reference [10], and microprocessors to PCs and FPGAs, as it is presented in Reference [21].

As future work, it is left for further research on use of multivalued piecewise linear function formulation, presented in Reference [51], which could generate two research lines. One for multiple-target planning of robots with displacement in 3-D or more dimensions like: drones, underwater robots, computer animation, robot manipulators, and molecular simulations [3,8,46]. The other one, employing multivalued or parametrized piece-wise linear functions like the ones presented in Reference [61,62], could enhance the performance of MGHHPP_VG in terms of path length, as it can be deducted from the cases studies, which would allow obtaining paths using visibility points in forward and backward movement on both axis. Besides, further work is needed to extend HPPM and MTHPPM to handle mechanical restrictions of non-holonomic robots, multi-agent systems, and path planning for dynamic environments with uncertainties. Finally, considering the advantages of the proposed homotopy based planner over SBP algorithms in terms of path length (close to the shortest path found by the best run of RRT*), execution time (between five and one hundred times faster than the median of SBP algorithms), and memory consumption (about a thousandth of that used by the SBP algorithms) makes of this a good choice to be implemented in practical applications. Besides, the linear relation between the number of obstacles and the complexity (in terms of memory and execution time) for homotopic planners, as it was presented in References [10,21] against the exponential complexity increase of SBP algorithms [2,3,8,9,63,64,65] allows to conclude that MTHPPM is the best option to planning collision-free robot motion paths. Especially, when it is needed to be implemented in a system with limited resources (like embedded systems) or required to solve complex environments where time constraints are tight.

## Figures and Tables

**Table 1:** Main characteristics of collision-free path planners.

Planner	Query	Completeness	Approach	Main Issue/Bottleneck
RRT [3,7,31]	Single	Probabilistically	Sampling-based	Collision query
Bi-RRT [32]	Single	Probabilistically	Sampling-based	Collision query
RRT* [7]	Single	Probabilistically	Sampling-based	Collision query
PRM [7,29]	Multiple	Probabilistically	Sampling-based	Collision query
EST [30]	Single	Probabilistically	Sampling-based	Collision query
KPIECE [1]	Single	Probabilistically	Sampling-based	Collision query
LazyRRT [33]	Single	Probabilistically	Sampling-based	Density of nodes
LazyPRM [34]	Multiple	Probabilistically	Sampling-based	Density of nodes
APF [16,17,18]	Single	Semi-complete	Potential fields	Local minima
A* [24,25,26]	Single	Resolution	Cells decomposition	Grid resolution
Visibility graphs [12,13,14,15]	Multiple	Complete	Graph-based	Obstacles density
HPPM [10,19,20]	Single	Semi-complete	HCM (Mathematical model)	Repulsion parameter selection

**Table 2:** Number of reversal effects and non-convergences solved in SA for HPPM and MTHPPM, case studies 1–4, and using DST technique.

Methodology		Non-Convergence Solved in SA (# Step)	Reversal Effects Solved in SA (# Step)	Total Steps
**Case 1**	HPPM	0	1940,1986,2974	3033
	MTHPPM_VG	1257,1927,1989,2373,2471,2492,2549	1825,1984,1994,2036	2602
**Case 2**	HPPM	1303	0	3594
	MTHPPM_VG	1248,1984,2012,2061, 2417,2441,2483,2558,2636	1318,1579,1908,1961 2018,2103,2457,2636	2689
**Case 3**	HPPM	0	0	3633
	MTHPPM_VG	1427,1491,2288,2316,2610	1777,2611	3192
**Case 4**	HPPM	17	36	2437
	MTHPPM_VG	17,210,245,611	565	2046

**Table 3:** Summarized performance results for case studies 1–4. Metrics: memory consumption (M), execution time (T), and length of path (L).

Planner	Metric	Case 1			Case 2			Case 3			Case 4		
		Min	Med	Max	Min	Med	Max	Min	Med	Max	Min	Med	Max
**EST**	M(MB)	69.3	69.5	69.5	66.2	66.4	66.4	39.3	39.4	39.4	49	49	49
	T(s)	1.13	3.10	20.0	1.21	4.16	20.0	0.86	1.53	2.93	0.619	1.629	8.613
	L	0.613	2.679	4.219	2.002	2.711	3.418	2.769	3.650	4.764	2.658	3.677	5.057
**KPIECE1**	M(MB)	69.5	69.5	69.5	66.4	66.4	66.4	39.4	39.4	39.4	49	49	49
	T(s)	1.30	2.84	7.16	1.0	7.3	20.0	1.53	3.78	11.7	1.41	4.09	15.3
	L	2.969	4.545	6.847	3.147	4.749	6.563	4.235	6.074	7.834	3.795	6.340	9.955
**PRM**	M(MB)	69.5	69.9	70	66.7	66.8	66.8	39.7	39.9	40.0	49.2	49.3	49.3
	T(s)	20.0	20.0	20.1	20.00	20.10	20.18	20.0	20.1	20.2	20.0	20.0	20.1
	L	1.679	1.792	2.128	1.746	1.904	2.195	2.380	2.508	2.690	2.123	2.228	2.502
**RRT**	M(MB)	70.0	80.0	80.3	66.8	70.1	70.1	42.5	49.5	51.3	50.07	64	64.25
	T(s)	1.28	5.34	20.0	5.010	19.49	20.11	7.44	11.6	20.0	15.7	20.0	20.1
	L	1.824	1.971	2.293	1.626	2.004	2.488	2.666	2.861	3.144	1.382	1.620	3.463
**Bi-RRT**	M(MB)	80.3	80.3	80.3	70.1	74.0	74.0	51.3	52.4	52.4	64.2	67.5	68.1
	T(s)	0.37	1.14	10.59	0.925	3.368	20.07	3.27	7.09	15.4	5.91	11.2	20.1
	L	1.802	1.949	2.554	1.879	2.049	2.246	2.668	2.836	3.037	2.577	2.930	3.837
RRT*	M(MB)	80.3	80.5	80.5	74.0	76.4	76.4	52.4	52.4	52.4	68.3	68.3	68.3
	T(s)	20.0	20.0	20.1	20.00	20.07	20.14	20.0	20.0	20.1	20.0	20.0	20.1
	L	1.237	1.587	1.735	1.058	1.671	2.006	1.458	2.266	2.337	1.140	2.028	2.156
**LazyPRM**	M(MB)	69.2	87.2	97.1	69.9	103.1	123.9	43.4	64.2	67.9	51	81.4	82.2
	T(s)	20.0	20.0	23.5	20.02	20.07	20.33	20.0	20.0	20.1	20.0	20.0	20.1
	L	1.633	1.687	1.758	1.728	1.789	1.946	2.337	2.393	2.477	2.081	2.159	2.225
**LazyRRT**	M(MB)	97.1	97.1	98.0	123.9	123.9	123.9	67.9	68.4	68.9	50.0	64	64.25
	T(s)	1.06	8.43	20.14	2.825	13.41	20.57	4.16	18.4	20.2	15.79	20.07	20.11
	L	1.851	2.200	2.940	1.894	2.286	2.589	2.734	3.116	3.783	1.382	1.620	3.463
**MTHPPM_VG**	M(MB)	0.075			0.0765			0.08186			0.056807		
	T(s)	0.19			0.23397			0.159609			0.255962		
	L	1.654			1.851			2.708			2.058		
**HPPM**	M(MB)	0.085			0.0984			0.08972			0.06555		
	T(s)	0.22			0.30011			0.122805			0.247262		
	L	2.098			2.637			2.699			2.407		

## References

[1] Şucan I.A., Kavraki L.E. (2012). A Sampling-Based Tree Planner for Systems with Complex Dynamics. IEEE Trans. Robot..

[2] Elbanhawi M., Simic M. (2014). Sampling-Based Robot Motion Planning: A Review. IEEE Access.

[3] LaValle S.M. (2006). Planning Algorithms.

[4] Kalamian N., Niri M.F., Mehrabizadeh H. Design of a Suboptimal Controller based on Riccati Equation and State-dependent Impulsive Observer for a Robotic Manipulator. Proceedings of the 2019 6th International Conference on Control, Instrumentation and Automation (ICCIA).

[5] Xunyu Z., Jun T., Huosheng H., Xiafu P. (2020). Hybrid Path Planning Based on Safe A* Algorithm and Adaptive Window Approach for Mobile Robot in Large-Scale Dynamic Environment. J. Intell. Robot. Syst..

[6] Patle B.K., Pandey A., Parhi D., Jagadeesh A. (2019). A review: On path planning strategies for navigation of mobile robot. Def. Technol..

[7] Karaman S., Frazzoli E. (2011). Sampling-based Algorithms for Optimal Motion Planning. Int. J. Rob. Res..

[8] Al-Bluwi I., Siméon T., Cortés J. (2012). Motion planning algorithms for molecular simulations: A survey. Comput. Sci. Rev..

[9] Kleinbort M., Salzman O., Halperin D. (2016). Collision Detection or Nearest-Neighbor Search? On the Computational Bottleneck in Sampling-Based Motion Planning. CoRR.

[10] Diaz-Arango G., Vázquez-Leal H., Hernandez-Martinez L., Pascual M.T.S., Sandoval-Hernandez M. (2017). Homotopy Path Planning for Terrestrial Robots Using Spherical Algorithm. IEEE Trans. Autom. Sci. Eng..

[11] Sharma K., Doriya R. (2020). Path planning for robots: An elucidating draft. Int. J. Intell. Robot. Appl..

[12] Nguyet T.T.N., Hoai T.V., Thi N.A. Some Advanced Techniques in Reducing Time for Path Planning Based on Visibility Graph. Proceedings of the 2011 Third International Conference on Knowledge and Systems Engineering.

[13] Tran N., Nguyen D.T., Vu D.L., Truong N.V. Global path planning for autonomous robots using modified visibility-graph. Proceedings of the 2013 International Conference on Control, Automation and Information Sciences (ICCAIS).

[14] Jan G.E., Sun C.C., Tsai W.C., Lin T.H. (2014). An *O*(*n**l**o**g**n*) Shortest Path Algorithm Based on Delaunay Triangulation. IEEE/ASME Trans. Mechatron..

[15] Foskey M., Garber M., Lin M.C., Manocha D. A Voronoi-based hybrid motion planner. Proceedings of the 2001 IEEE/RSJ International Conference on Intelligent Robots and Systems, Expanding the Societal Role of Robotics in the the Next Millennium (Cat. No.01CH37180).

[16] Lee M.C., Park M.G. (2003). Artificial potential field based path planning for mobile robots using a virtual obstacle concept. Adv. Intell. Mechatron. IEEE/ASME Int. Conf..

[17] Laue T., Rofer T. (2005). A behavior architecture for autonomous mobile robots based on potential fields. RoboCup 2004: Robot Soccer World Cup VIII.

[18] Rimon E., Koditschek D.E. (1992). Exact robot navigation using artificial potential functions. IEEE Trans. Robot. Autom..

[19] Vazquez-Leal H., Marin-Hernandez A., Khan Y., Yildirim A., Filobello-Nino U., Castaneda-Sheissa R., Jimenez-Fernandez V. (2013). Exploring collision-free path planning by using homotopy continuation methods. Appl. Math. Comput..

[20] Diaz-Arango G., Sarmiento-Reyes A., Hernandez-Martinez L., Vazquez-Leal H., Lopez-Hernandez D.D., Marin-Hernandez A. Path optimization for terrestrial robots using Homotopy Path Planning Method. Proceedings of the 2015 IEEE International Symposium on Circuits and Systems (ISCAS).

[21] De Cos-Cholula H.E., Diaz-Arango G.U., Hernandez-Martinez L., Vazquez-Leal H., Sarmiento-Reyes A., Sanz-Pascual M.T., Herrera-May A.L., Castaneda-Sheissa R. (2020). FPGA Implementation of Homotopic Path Planning Method with Automatic Assignment of Repulsion Parameter. Energies.

[22] Wang H., Yu Y., Yuan Q. Application of Dijkstra algorithm in robot path-planning. Proceedings of the 2011 Second International Conference on Mechanic Automation and Control Engineering.

[23] Koziol S., Hasler P., Stilman M. Robot path planning using Field Programmable Analog Arrays. Proceedings of the 2012 IEEE International Conference on Robotics and Automation.

[24] Kopřiva S., Šišlák D., Pavlíček D., Pěchouček M. Iterative accelerated A* path planning. Proceedings of the 49th IEEE Conference on Decision and Control (CDC).

[25] Soltani A., Tawfik H., Goulermas J., Fernando T. (2002). Path planning in construction sites: Performance evaluation of the Dijkstra, A*, and GA search algorithms. Adv. Eng. Inform..

[26] Saian P.O.N., Suyoto, Pranowo Optimized A-Star algorithm in hexagon-based environment using parallel bidirectional search. Proceedings of the 2016 8th International Conference on Information Technology and Electrical Engineering (ICITEE).

[27] Qiang L., Haibao W., Yan Z., Jingchang H. (2019). Research on path planning of mobile robot based on improved ant colony algorithm. Neural Comput. Appl..

[28] Reinoso O., Wu S., Du Y., Zhang Y. (2020). Mobile Robot Path Planning Based on a Generalized Wavefront Algorithm. Math. Probl. Eng. Hindawi.

[29] Kavraki L.E., Svestka P., Latombe J.C., Overmars M.H. (1996). Probabilistic roadmaps for path planning in high-dimensional configuration spaces. IEEE Trans. Robot. Autom..

[30] Hsu D., Latombe J.C., Motwani R. Path planning in expansive configuration spaces. Proceedings of the International Conference on Robotics and Automation.

[31] LaValle S.M. (1998). Rapidly-Exploring Random Trees: A New Tool for Path Planning. http://citeseerx.ist.psu.edu/viewdoc/summary?doi=10.1.1.35.1853.

[32] Kuffner J.J., LaValle S.M. RRT-Connect: An efficient approach to single-query path planning. Proceedings of the 2000 ICRA. Millennium Conference. IEEE International Conference on Robotics and Automation. Symposia Proceedings (Cat. No.00CH37065).

[33] Hauser K. Lazy collision checking in asymptotically-optimal motion planning. Proceedings of the 2015 IEEE International Conference on Robotics and Automation (ICRA).

[34] Bohlin R., Kavraki L.E. Path planning using lazy PRM. Proceedings of the 2000 ICRA. Millennium Conference, IEEE International Conference on Robotics and Automation, Symposia Proceedings (Cat. No.00CH37065).

[35] Wang W., Li Y., Xu X., Yang S.X. An adaptive roadmap guided Multi-RRTs strategy for single query path planning. Proceedings of the 2010 IEEE International Conference on Robotics and Automation.

[36] Diaz-Arango G., Hernandez-Martinez L., Sarmiento-Reyes A., Vazquez-Leal H. Fast and robust homotopy path planning method for mobile robotics. Proceedings of the 2016 IEEE International Symposium on Circuits and Systems (ISCAS).

[37] Cos-Cholula H.E.D., Díaz-Arango G.U., Hernández-Martínez L., Sarmiento-Reyes A. An Homotopy Path Planning Method with automatic fixed value assignation of repulsion parameter for mobile robotics. Proceedings of the 2016 13th International Conference on Electrical Engineering, Computing Science and Automatic Control (CCE).

[38] Park M.G., Lee M.C. (2003). A new technique to escape local minimum in artificial potential field based path planning. KSME Int. J..

[39] Matoui F., Boussaid B., Abdelkrim M.N. Local minimum solution for the potential field method in multiple robot motion planning task. Proceedings of the 2015 16th International Conference on Sciences and Techniques of Automatic Control and Computer Engineering (STA).

[40] Luo C., Mo H., Shen F., Zhao W. (2016). Multi-Goal Motion Planning of an Autonomous Robot in Unknown Environments by an Ant Colony Optimization Approach.

[41] Hernandez K., Bacca B., Posso B. (2017). Multi-goal Path Planning Autonomous System for Picking up and Delivery Tasks in Mobile Robotics. IEEE Lat. Am. Trans..

[42] Bueckert J., Yang S.X., Yuan X., Meng M.Q.H. Neural dynamics based multiple target path planning for a mobile robot. Proceedings of the 2007 IEEE International Conference on Robotics and Biomimetics (ROBIO).

[43] Devaurs D., Siméon T., Cortés J. A multi-tree extension of the transition-based RRT: Application to ordering-and-pathfinding problems in continuous cost spaces. Proceedings of the 2014 IEEE/RSJ International Conference on Intelligent Robots and Systems.

[44] Faigl J., Váňa P., Deckerová J. (2019). Fast Heuristics for the 3-D Multi-Goal Path Planning Based on the Generalized Traveling Salesman Problem with Neighborhoods. IEEE Robot. Autom. Lett..

[45] Ishida S., Rigter M., Hawes N. Robot Path Planning for Multiple Target Regions. Proceedings of the 2019 European Conference on Mobile Robots (ECMR).

[46] Petereit J., Emter T., Frey C.W. Safe mobile robot motion planning for waypoint sequences in a dynamic environment. Proceedings of the 2013 IEEE International Conference on Industrial Technology (ICIT).

[47] Yamamura K. (1992). Simple algorithms for tracing solution curves. IEEE Int. Symp. Circuits Syst..

[48] Torres-Muñoz D., Vazquez-Leal H., Hernandez-Martinez L., Sarmiento-Reyes A. (2014). Improved spherical continuation algorithm with application to the double-bounded homotopy (DBH). Comput. Appl. Math..

[49] Oliveros-Munoz J.M., Jiménez-Islas H. (2013). Hyperspherical path tracking methodology as correction step in homotopic continuation methods. Chem. Eng. Sci..

[50] Ramirez-Pinero A., Vazquez-Leal H., Jimenez-Fernandez V.M., Sedighi H.M., Rashidi M.M., Filobello-Nino U., Castaneda-Sheissa R., Huerta-Chua J., Sarmiento-Reyes L.A., Laguna-Camacho J.R. (2016). Speed-up hyperspheres homotopic path tracking algorithm for PWL circuits simulations. SpringerPlus.

[51] Chua L.O., Kang S.M. (1977). Section-wise piecewise-linear functions: Canonical representation, properties, and applications. Proc. IEEE.

[52] Chua L., Deng A.C. (1986). Canonical piecewise-linear modeling. IEEE Trans. Circuits Syst..

[53] Julian P., Desages A., Agamennoni O. (1999). High-level canonical piecewise linear representation using a simplicial partition. IEEE Trans. Circuits Syst. Fundam. Theory Appl..

[54] Julian P., Desages A., D’Amico B. (2000). Orthonormal high-level canonical PWL functions with applications to model reduction. IEEE Trans. Circuits Syst. Fundam. Theory Appl..

[55] Guzelis C., Goknar I.C. (1991). A canonical representation for piecewise-affine maps and its applications to circuit analysis. IEEE Trans. Circuits Syst..

[56] Schmidt M., Fung G., Rosales R., Kok J.N., Koronacki J., Mantaras R.L.d., Matwin S., Mladenič D., Skowron A. (2007). Fast Optimization Methods for L1 Regularization: A Comparative Study and Two New Approaches. Machine Learning: ECML 2007.

[57] Sucan I.A., Moll M., Kavraki L.E. (2012). The Open Motion Planning Library. IEEE Robot. Autom. Mag..

[58] Moll M., Şucan I.A., Kavraki L.E. (2015). Benchmarking Motion Planning Algorithms: An Extensible Infrastructure for Analysis and Visualization. IEEE Robot. Autom. Mag..

[59] Coulter R.C. (1992). Implementation of the Pure Pursuit Path Tracking Algorithm.

[60] Morales J., Martínez J.L., Martínez M.A., Mandow A. (2009). Pure-Pursuit Reactive Path Tracking for Nonholonomic Mobile Robots with a 2D Laser Scanner. EURASIP J. Adv. Signal Process..

[61] Jimenez-Fernandez V.M., Jimenez-Fernandez M., Vazquez-Leal H., Filobello-Nino U.A., Castro-Gonzalez F.J. (2016). Smoothing the High Level Canonical Piecewise-Linear Model by an Exponential Approximation of its Basis-Function. Comput. Sist..

[62] Jimenez-Fernandez V.M., Jimenez-Fernandez M., Vazquez-Leal H., Muñoz-Aguirre E., Cerecedo-Nunez H.H., Filobello-Nino U.A., Castro-Gonzalez F.J. (2016). Transforming the canonical piecewise-linear model into a smooth-piecewise representation. SpringerPlus.

[63] Saha M., Latombe J.C., Chang Y.C., Prinz F. (2005). Finding Narrow Passages with Probabilistic Roadmaps: The Small-Step Retraction Method. Auton. Robot..

[64] Sun Z., Hsu D., Jiang T., Kurniawati H., Reif J.H. (2005). Narrow passage sampling for probabilistic roadmap planning. IEEE Trans. Robot..

[65] Zhong J., Su J. Narrow passages identification for Probabilistic Roadmap Method. Proceedings of the 30th Chinese Control Conference.

